# Expression of multiple horizontally acquired genes is a hallmark of both vertebrate and invertebrate genomes

**DOI:** 10.1186/s13059-015-0607-3

**Published:** 2015-03-13

**Authors:** Alastair Crisp, Chiara Boschetti, Malcolm Perry, Alan Tunnacliffe, Gos Micklem

**Affiliations:** Department of Chemical Engineering and Biotechnology, University of Cambridge, New Museums Site, Pembroke Street, Cambridge, CB2 3RA UK; Department of Genetics, University of Cambridge, Cambridge, CB2 3EH UK; Cambridge Systems Biology Centre, University of Cambridge, Cambridge, CB2 1QR UK

## Abstract

**Background:**

A fundamental concept in biology is that heritable material, DNA, is passed from parent to offspring, a process called vertical gene transfer. An alternative mechanism of gene acquisition is through horizontal gene transfer (HGT), which involves movement of genetic material between different species. HGT is well-known in single-celled organisms such as bacteria, but its existence in higher organisms, including animals, is less well established, and is controversial in humans.

**Results:**

We have taken advantage of the recent availability of a sufficient number of high-quality genomes and associated transcriptomes to carry out a detailed examination of HGT in 26 animal species (10 primates, 12 flies and four nematodes) and a simplified analysis in a further 14 vertebrates. Genome-wide comparative and phylogenetic analyses show that HGT in animals typically gives rise to tens or hundreds of active ‘foreign’ genes, largely concerned with metabolism. Our analyses suggest that while fruit flies and nematodes have continued to acquire foreign genes throughout their evolution, humans and other primates have gained relatively few since their common ancestor. We also resolve the controversy surrounding previous evidence of HGT in humans and provide at least 33 new examples of horizontally acquired genes.

**Conclusions:**

We argue that HGT has occurred, and continues to occur, on a previously unsuspected scale in metazoans and is likely to have contributed to biochemical diversification during animal evolution.

**Electronic supplementary material:**

The online version of this article (doi:10.1186/s13059-015-0607-3) contains supplementary material, which is available to authorized users.

## Background

The acquisition of genes from an organism other than a direct ancestor (that is, horizontal gene transfer (HGT) also called lateral gene transfer) is well known in bacteria and unicellular eukaryotes, where it plays an important role in evolution [1], with recent estimates suggesting that on average 81% of prokaryotic genes have been involved in HGT at some point [2]. However, relatively few cases have been documented in multicellular organisms [3-7]. Reports of HGT in animals are usually limited to the description of the transfer of only one or a few genes, making the extent of horizontal gene transfer in animals unclear. Examples include the transfer of fungal genes for carotenoid biosynthesis to the pea aphid, which results in a red pigmentation and is thought to be beneficial to the aphid [8] and the transfer of a cysteine synthase from a bacterium into the arthropod lineage (likely two independent transfers into a phytophagous mite ancestor and a lepidopteran ancestor), which allows the detoxification of cyanide produced by host plants [9]. This activity is also found in nematodes, where it may have been acquired by HGT from plants [9]. Other examples of putatively adaptive HGT have been characterised in plant-parasitic nematodes, which produce cell-wall degrading enzymes from a number of horizontally transferred genes [3], and the coffee berry borer beetle, where a mannanase has been transferred from bacteria allowing the hydrolysation of coffee berry galactomannan [10].

In exceptional cases, high levels of HGT in animals have been reported, but this has been attributed to the lifestyles of the recipient organisms. For example, in bdelloid rotifers, which are desiccation-tolerant asexuals, up to approximately 10% of transcripts derive from horizontally acquired genes [11-13]. Desiccation results in both DNA breakage [14,15] and loss of membrane integrity (reviewed in [16]), both of which may potentiate HGT. Another unusual example is the transfer of the entire genome (>1 Mb) of the bacterium *Wolbachia* into the fruit fly *Drosophila ananassae*, although relatively few *Wolbachia* genes are transcribed in this case [17]. Genes from *Wolbachia* are frequently transferred to invertebrates [17,18], probably because the long-term association (either parasitic or mutualistic) between the bacterium and its hosts maintains their genomes in close proximity. Furthermore, as *Wolbachia* frequently infects the testes and ovaries of its hosts, it has access to their germlines, a prerequisite for the transmission of the acquired genes to the next generation.

These studies have led to the perception that HGT occurs very infrequently in most animals, especially in vertebrates [5,6]. Furthermore, there are concerns over the validity of the examples of HGT reported in humans [19-22]. The original report on the human genome sequence [19] described prokaryote-to-vertebrate HGT discovered by aligning human sequences to those of a small number of species (not many genomes were available at the time), including only two metazoans, *D. melanogaster* and *Caenorhabditis elegans*. Any proteins aligning to bacteria but not to these two metazoans, or to the other two eukaryotic proteomes used (*Arabidopsis thaliana* and *Saccharomyces cerevisiae*), were considered to be a result of prokaryote-to-vertebrate HGT. However, these four eukaryotic species do not contain orthologs of all ‘native’ human genes (that is, those not horizontally acquired), leading to incorrect identification of HGT (false positives) and the subsequent rejection of many cases by phylogenetic analyses [20-22]. The problem (the availability of a limited number of eukaryotic genomes for comparison in studies of HGT) has lessened in the intervening decade; thousands of proteomes (including several primates) are now available in UniProt, allowing prediction of HGT using alignment to hundreds of species and subsequent phylogenetic validation, as shown in recent work in invertebrates (for example, [12,23,24]). In the human, however, there have been no follow-up studies since the original genome paper, and the true scale of HGT in humans, and metazoans generally, remains unclear.

To remedy this, we initially identified non-metazoan to metazoan HGT in multiple *Drosophila*, *Caenohabditis* and primate (including human) species. Due to the controversy surrounding the human studies [19-22], we then took our analysis a step further by comparing multiple closely related species and combining information on horizontally transferred (‘foreign’) genes found in more than one species in the group, thereby reducing mis-identification of HGT caused by spurious alignments. In this way, we identified up to hundreds of active foreign genes in animals, including humans, suggesting that HGT provides important contributions to metazoan evolution.

## Results

### *Drosophila* species, *Caenorhabditis* species and primates have up to hundreds of active foreign genes

**Figure 1:**
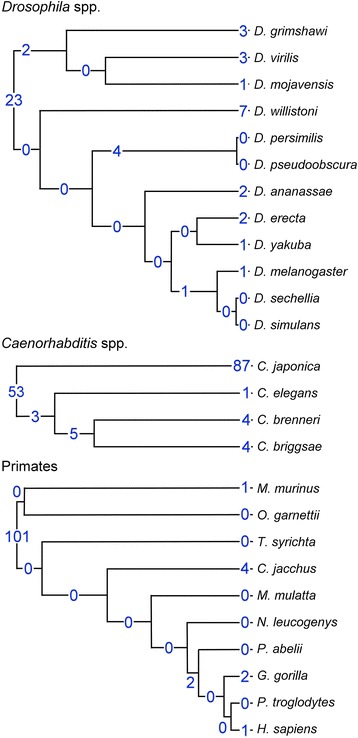
**Phylogenetic relationships of the main taxonomic groups studied.** The blue numbers indicate the ortholog groups mapping to each branch (HGT events). Events may have occurred anywhere along the branch, not just where the number is indicated. Events found at the base of the tree have occurred anywhere between the origin of the phylum and the base of the tree. Trees are not drawn to scale with each other.

To determine the scale of HGT across well-characterised taxonomic groups, we examined 12 *Drosophila* species, four *Caenorhabditis* species and 10 primates (Figure 1) for which high quality genomes and transcriptomes are available. For each transcribed gene, we calculated the HGT index, *h* (the difference between the bitscores of the best non-metazoan and the best metazoan matches), which gives a relative quantitative measure of how well a given gene aligns to non-metazoan versus metazoan sequences, with positive numbers indicating a better alignment to non-metazoan sequences [12]. For example, the *C. elegans* gene gut-obstructed 1 (*gob-1*), which encodes a trehalose-6-phosphate phosphatase, has a best non-metazoan match with a bitscore of 135 and a best metazoan match with a bitscore of 39.3 resulting in an HGT index of 95.7. As we were interested in more than just very recent HGT, we excluded members of the test species’ phylum from the metazoan matches. This allowed us to identify HGT over evolutionary periods encompassing hundreds of millions of years, as opposed to only identifying HGT that occurred since the test species’ divergence from its most closely related species (likely up to tens of millions of years). Hereafter, when we refer to matches to metazoan sequences, we mean these subsets.

**Figure 2:**
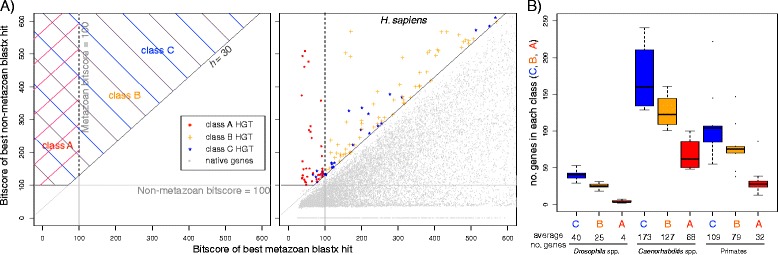
**HGT genes by class. (** **A** **)** The left panel shows a schematic representation of the HGT classes: class B and C genes have *h* index ≥ 30 and bitscore of the best non-metazoan blastx hit ≥ 100 (they are distinguished by *h*_*orth*_, which is not shown on this figure), while class A genes must additionally have bitscore <100 for the best metazoan blastx hit. The right panel shows the scores for all genes in *H. sapiens*, colour-coded according to their classification (class A: red, class B: orange, class C: blue, native genes: grey). **(** **B** **)** Box-plot of the number of genes in each class, for the three main taxa analysed (*Drosophila* spp. *Caenorhabditis* spp., primates species), colour-coded according to the same scheme (class A: red, class B: orange, class C: blue).

We first identified a base level of HGT (called class C) by using conservative thresholds of *h* ≥30 (as in [12]) (meaning that the gene aligns much better, and is therefore much more similar, to non-metazoan genes) and bitscore of best non-metazoan match ≥100 (thereby excluding bad alignments to non-metazoans). The example given above (*gob-1*) passes these thresholds and is therefore at least class C HGT. This per-species information was then combined for each taxon (*Drosophila*, *Caenorhabditis* and primates) to construct ortholog groups. For each ortholog group we calculated the average *h* value of all members (*h*_*orth*_) and defined the genes with *h*_*orth*_ ≥30 as class B, a subset of class C. These genes are, on average, predicted as HGT in all tested species they are found in. The gene *gob-1* has homologs in *C. brenneri*, *C. briggsae* and *C. japonica*, with values of *h* = 102, *h* = 97.1 and *h* = 86.4 respectively, giving an average *h* (*h*_*orth*_) of 95.3 and as such *gob-1* (ands its homologs) are also class B HGT. Finally, we applied a still more stringent filter to define class A foreign genes (a subset of class B), which had only very poor alignments to metazoan sequences and whose orthologs, as used to define class B, also had similarly poor alignments to metazoan sequences. To do this, we identified those sequences whose best match to a metazoan had a bitscore <100 and whose ortholog groups contain no genes with metazoan matches of bitscore ≥100 (Figure 2A). The gene *gob-1* has no metazoan matches with bitscore ≥100 (best metazoan match = 39.3) and the same is true for its homologs (best matches of 37, 38.9 and 36.6, respectively), as such it is also class A HGT.

We then performed phylogenetic analyses for all genes of each of the above classes and found that an average of 55% of all class C genes, 65% of all class B genes and 88% of all class A genes were phylogenetically validated as foreign. This validation and further manual analysis (Additional files 1 and 2) suggested that, while false positives are minimised as C → B → A, some true positives are also lost. Therefore, class A genes represent a minimum estimate of the level of HGT for a given species.

We found that *Caenorhabditis* species have, on average, 173, 127 and 68 genes in HGT classes C, B and A, respectively. In contrast, *Drosophila* species have fewer active foreign genes with, on average, 40 genes in class C, 25 in class B, and only four in class A. Primate HGT levels fall between those of the invertebrate taxa, with an average of 109, 79 and 32 genes per species in classes C, B and A, respectively (Figure 2B, Additional files 2 and 3).

### Identified foreign genes are unlikely to be explained by alternative hypotheses

To verify that the foreign genes we identified do indeed belong to the species under study and are not contamination (this is a problem in a number of animal genome sequences; see ‘Phylogenetic validation’ in Additional file 1), we tested whether they were found on the same genomic scaffolds as (that is, were linked to) genes of metazoan origin (native genes). Across all species we found an average of only nine class C genes per species (6.6% of foreign genes) that were not linked to native genes (Additional file 2), with correspondingly low proportions for class B and A genes. Demonstration of such high levels of linkage was only possible due to the high quality of the assemblies of these genomes. Although most species showed a high degree of linkage, there were three outliers (see Additional file 1), but even if all unlinked genes were contamination, which is not necessarily the case, this would have a minimal impact on the levels of HGT detected.

An alternative hypothesis to explain our data is that the genes we label as foreign in any single species are actually the result of conventional vertical descent, but have been lost in all other animal lineages. The parsimony principle tells us that we should choose the simplest explanation, which might be determined by comparing the rate of gene loss and the rate of gene gain by HGT. However, while the rate of gene loss over time can be estimated, at this point we cannot accurately estimate the rate of HGT over anything less than the time since the common ancestor of all metazoans, due to limited data. The rates that should actually be compared are the rates of gene loss and HGT at the earliest branches of the eukaryotic tree, but these rates are especially difficult to determine as the very long periods of time involved mean that ortholog determination (necessary to find which genes have been lost/gained) is hard. Furthermore, published estimates of the rate of gene loss typically treat all genes as equal, but the actual rate varies between types of genes and types of organisms (for example, parasites have higher loss rates [25,26]). As HGT involves the transfer of only a subset of genes (see section ‘Many horizontally acquired genes code for enzyme activities’, below), a generic gene loss rate is not comparable to the HGT rate.

Given these difficulties we attempted to differentiate between the two hypotheses with a different method. We looked at the functions of foreign genes and compared them to those of native genes that are known to have been lost in all other animal lineages, but were not predicted as foreign (genes for which the alternate hypothesis is true) and found significant differences between the foreign genes we identified and native genes fulfilling these criteria (see section ‘Many horizontally acquired genes code for enzyme activities’, below). Therefore, while we cannot entirely discount the gene loss hypothesis, it seems an unlikely explanation for the tens or hundreds of foreign genes per genome that we observe.

### Identification of new foreign genes and confirmation of previously reported examples

The first report of the human genome sequence highlighted 223 protein sequences (of which 113 were confirmed as present in the genome by PCR) that were proposed to originate from bacteria by HGT [19]. While some of these genes were later confirmed as foreign, many were rejected [20-22]. At the time of writing, it is difficult to assess all of these sequences because some early identifiers have not been maintained, but we have been able to confirm or reclaim 17 previously reported examples as foreign (some also confirmed by other studies; Additional file 4). Furthermore, we identified up to 128 additional foreign genes in the human genome (128 class C, of which 93 are class B and 33 class A), giving a total of 145 class C genes, of which 110 are class B and 39 class A.

**Figure 3:**
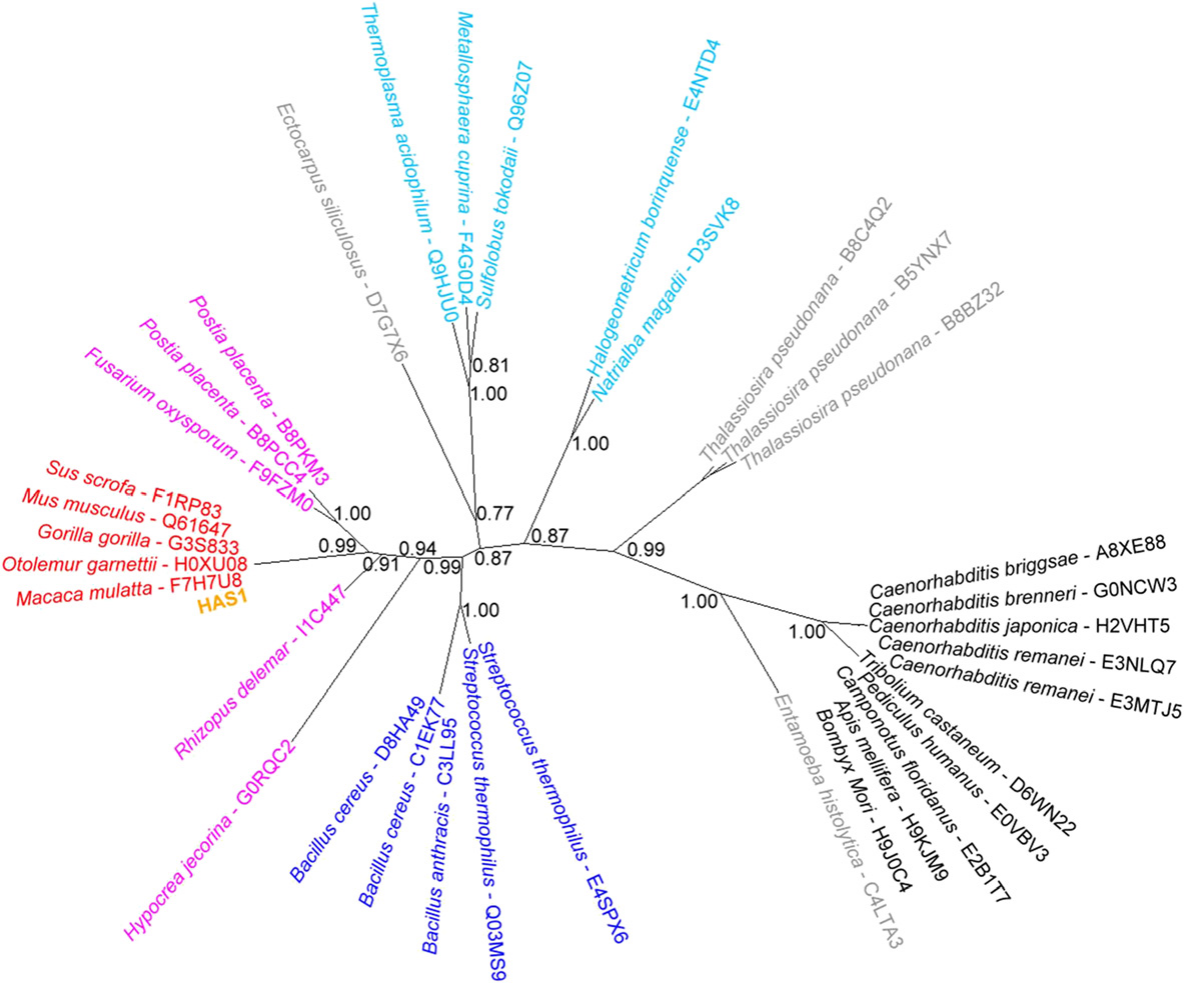
**Phylogenetic tree for the human gene HAS1.** For each branch the species name and UniProt accession is shown. The human gene under analysis is shown in orange, proteins from chordates are in red, other metazoa in black, fungi in pink, plants in green, protists in grey, archaea in light blue and bacteria in dark blue. Numbers indicate aLRT support values for each branch where higher than 0.75 (on short terminal branches the support values are not shown).

Among these examples, we reclaim those encoding the hyaluronan synthases (HAS1-3). These were originally proposed as examples of prokaryote-to-metazoan HGT [19], but later rejected [20]; however, neither study considered foreign taxa other than bacteria. We were able to identify all three hyaluronan synthases as class A HGT, originating from fungi, an assessment supported by our phylogenetic analysis (Figure 3). The HAS genes appear in a wide variety of chordates, but not in non-chordate metazoans, suggesting they result from the transfer of a single gene around the time of the common ancestor of Chordata, before undergoing duplications to produce the three genes found in primates. As the original rebuttal paper [20] only focused on recent HGT, and did not look for eukaryotic matches outside Chordata, they could not detect this ancient HGT.

We also identify cases of HGT reported more recently that have not been analysed in detail despite the potentially interesting consequences of such a finding. For example, the fat mass and obesity associated gene (FTO, in Additional file 5: Figure S1A) seems to be present only in marine algae and vertebrates [27,28], which is a highly unusual distribution. Another gene proposed to have been horizontally transferred is the ABO blood group gene (ABO, in Additional file 5: Figure S1B), which is suggested to enhance mutualism between vertebrates and bacteria [29]. We identified both these genes as class A HGT with phylogenetic validation (Additional file 3).

In the invertebrates, Dunning Hotopp *et al.* [17] reported the horizontal transfer of nearly the entire *Wolbachia* genome into the *D. ananassae* genome and the expression of 44 formerly *Wolbachia* genes, as evidenced by PCR amplification of their transcripts in flies that had been cured of *Wolbachia* and partial re-sequencing of the *D. ananassae* genome. These genes are still not included in the official genome of *D. ananassae*, likely because eukaryote genome sequencing projects routinely screen for and exclude bacterial sequences on the assumption that these represent contamination. Consequently, they were not in the dataset tested in this study and therefore do not appear among the foreign genes identified in *D. ananassae*. However, we did find one gene in *D. ananassae*, GF19976, which has not been identified previously as foreign and that may originate from *Wolbachia*.

Parkinson and Blaxter [30] identified four horizontally acquired genes in *C. elegans*. We identified all of these, three as class B HGT and the fourth as class A (highlighted in yellow in Additional file 3), but we also discovered a further 135 class C genes, of which 113 were class B and 71 class A (Additional file 3). This discrepancy with Parkinson and Blaxter [30] arises largely because these authors aligned *C. elegans* sequences with only a single non-metazoan (*S. cerevisiae*). Accordingly, we identified three of the four known foreign genes as fungal in origin, with the fourth also aligning well to fungal proteins (although we find it originated from a protist). Overall, however, only 4% to 15% of *C. elegans* HGT (depending on class) is of fungal origin (Additional file 2), with rather more (52% to 72%) deriving from bacteria (not assessed in ref. [30]). As mentioned in the Background section, there is phylogenetic evidence that the Cysteine Synthase Like genes found in nematodes, including *C. elegans* (*cysl1* - *4*), may have been acquired from plants [9]. Our analysis supports this conclusion with all four genes being class B HGT of plant origin and three being phylogenetically validated. HGT also occurs in a number of other nematodes, in particular the parasitic root-knot nematodes, in which as many as approximately 3% of all genes may be horizontally acquired [3,24].

### Many horizontally acquired genes code for enzyme activities

In prokaryotes, horizontally acquired genes tend to be operational, typically encoding enzymes, rather than informational, that is, genes involved in transcription and translation [31]. It has recently been suggested that network connectivity is a more important consideration than function [32], but nevertheless most identified foreign genes are concerned with metabolism. Consistent with this, 83% of foreign genes in the bdelloid rotifer encode enzymes [12]. To determine whether this applies to HGT throughout metazoans, we first inspected Gene Ontology (GO) terms that were found at unexpectedly high levels among class A foreign genes (‘enriched GO terms’), then determined which GO terms indicated enzyme activities, and finally calculated the percentage of enzyme activities for both enriched and un-enriched terms. In almost all *Caenorhabditis* and primate species, enriched GO terms were significantly more likely (chi-squared test: 3E-9 ≤ *P* ≤ 0.05; Additional file 2) to describe enzyme activities than un-enriched terms (on average, 42% of enriched terms relate to enzyme activities vs. 26% of un-enriched terms; Additional file 2). In *Drosophila* species, insufficient terms were enriched to perform the calculation. Enriched GO terms in class B genes were also more likely to relate to enzyme activities. The second largest group of foreign genes codes for membrane-bound proteins, another category of operational genes. Therefore, like in prokaryotes [22], HGT is biased towards operational genes in metazoans.

A possible alternative explanation for the genes suggested to result from HGT is that they are actually the product of vertical descent in the concerned species, but have been lost in all other animal lineages (as discussed above in ‘Identified foreign genes are unlikely to be explained by alternative hypotheses’). This explanation is more likely in the primates as their HGT is predominately ancient (see section ‘Horizontal gene transfer is both ancient and ongoing’ below), reducing the number of times the gene must be lost. To test this hypothesis, the same GO analysis was performed on native genes that are found in chordates and not in non-chordate metazoans (that is, genes that have been lost in all non-chordate metazoans; a possible alternative explanation for the putative foreign genes we identify). In all primate species, enriched GO terms for these genes (when compared to those from all other native genes) were significantly less likely (chi-squared test: *P* ≤0.05; Additional file 2) to describe enzyme activities than un-enriched terms (on average, 4% vs. 20%; Additional file 2). This is the opposite of the result for foreign genes, suggesting that an alternative hypothesis of gene loss does not explain our findings.

### Foreign gene functions

Many foreign genes are, like many native genes, currently uncharacterised, even in intensively studied model organisms; for example, the human (foreign) gene ENSG00000136830 is annotated ‘family with sequence similarity 129, member B’, but there is no information on its role. Where foreign genes have meaningful annotation, it is clear they code for a wide variety of different functions across a broad range of categories, some of which may overlap. Here we describe the six most noteworthy categories, from largest to smallest, across *C. elegans*, *D. melanogaster* and the human (Additional file 3).

In *C. elegans*, the largest category includes genes connected to the innate immune response (16 genes), including genes that specifically target bacterial cell walls (*lys* family), genes targeting fungi (for example, endochitinases) and other more general immune system genes (for example, *thn-3* and *thn-4*). The second largest category comprises eight genes involved in lipid metabolism, including the breakdown of fatty acids by beta-oxidation (for example, fatty acid CoA synthetase family, *acs-11*), as well fatty acid synthesis (for example, fatty acid desaturase, *fat-1*). The third category includes four genes involved in macromolecule modification, which encompasses activities such as phosphorylation, methylation and ubiquitination. The fourth category governs stress responses and includes a heat shock protein (*dnj-16*), an LEA protein (*lea-1*) and two genes involved in the trehalose synthesis pathway: trehalose-6-phosphate phosphatase (*gob-1*) and trehalose-phosphate synthase (*tps-1*). Trehalose production allows *C. elegans* dauer larvae to survive extreme desiccation [33], while LEA proteins are also linked to tolerance of water stress in *C. elegans* [34] and other invertebrates, as well as plants and microorganisms (reviewed in [35]). The fifth category consists of antioxidant activities (one gene; glutathione peroxidase, *gpx-2*) and the sixth category is amino-acid metabolism, also consisting of a single gene, coding for asparagine synthesis (*asns-2*).

There are far fewer foreign genes in *D. melanogaster*, but we do see genes belonging to some of the same categories as in *C. elegans*, namely macromolecule modification (two genes), the innate immune response (three genes) and stress response (three genes). The three *D. melanogaster* immune response genes all belong to the same family of proteins, which is involved in the phagocytosis of bacteria, while the three stress response genes are all involved in the trehalose synthesis pathway: two trehalose phosphatases (FBgn0031907, FBgn0031908) and a trehalose-phosphate synthase (Tps1). While this last gene has the same name as a *C. elegans* trehalose-phosphate synthase gene (Tps1/*tps-1*), alignment shows they are dissimilar, especially outside the catalytic domain, suggesting they do not originate from the same HGT event (in Additional file 5: Figure S2). Likewise the trehalose phosphatases are not conserved across species.

In the human we find genes in five of the six categories: amino-acid metabolism (two genes), macromolecule modification (15 genes), lipid metabolism (13 genes), antioxidant activities (five genes) and innate immune response (seven genes). The lipid metabolism genes include genes with similar functions to the *C. elegans* genes, such as the breakdown of fatty acids by beta-oxidation (for example, enoyl-CoA, hydratase/3-hydroxyacyl CoA dehydrogenase, EHHADH), as well as a wide variety of other functions including the formation of glycolipids via chain extension (for example, globoside alpha-1,3-N-acetylgalactosaminyltransferase 1, GBGT1) and transmembrane transporters required for lipid homestasis (for example, ATP-binding cassette, sub-family G (WHITE), member 5, ABCG5). The innate immune response genes include genes involved in the inflammatory response (for example, immunoresponsive 1 homolog, IRG1), genes for immune cell signalling (for example, phosphatidylinositol-4,5-bisphosphate 3-kinase, catalytic subunit gamma, PIK3CG) and antimicrobial genes (for example, epididymal peptidase inhibitor, EPPIN).

We do not find any of the same foreign genes in common across the three species because our method precludes this: such genes would have been present in a common ancestor and would be screened out as metazoan. However, we do find shared functions, such as the trehalose synthesis pathway in the invertebrates. Few genes are found in shared pathways. This may indicate that transfers happen one gene at a time, with each gene being separately integrated into the metabolic networks of the organism. Broadly speaking we do not see differences between the species in the functions encoded by foreign genes, except in the immune response category: the majority of the invertebrate genes encode enzymes that break down bacterial and fungal cell walls, which would seem to confer a clear adaptive advantage, while the human genes are more likely to code for signalling and regulation of the immune response and have less obvious advantages to the organism. This likely reflects the differences in age between the vertebrate and invertebrate HGT (see section ‘Horizontal gene transfer is both ancient and ongoing’, below), with the more recently acquired foreign genes in the invertebrates having a clearer role than the ancient foreign genes in the vertebrates, which have had longer to integrate into networks.

### Foreign genes predominately originate from bacteria and protists

**Figure 4:**
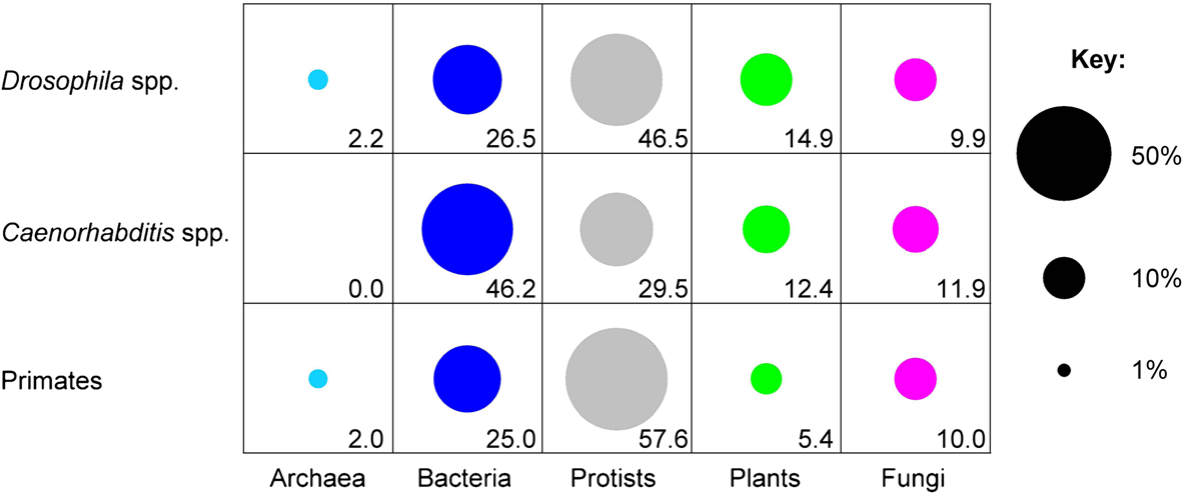
**Mean origin of class C foreign genes for each taxon.** Numbers show percentage contribution within each taxon (row). The same analyses for Class B or A genes show very similar patterns. The colour scheme is as in Figure 3: origin from archaea is light blue, from bacteria is dark blue, from protists is grey, from plants is green and from fungi is pink.

When calculating *h*, the likely taxon of origin of a foreign gene was taken to be the taxon of the best-matching protein. Bacteria and protists are the most common donors in all groups (Figure 4), which might reflect the relative abundance of the respective donor species in the environments of the recipient organisms. The phylogenetic validation of the foreign genes occasionally indicated a different origin than the original calculation (based on alignments and *h* index), but both methods agreed on average 92% of the time; performing the analysis shown in Figure 4 using phylogenetically predicted origins instead shows the same pattern of donors (data not shown). The identity of the actual donor species is much harder to determine, as the identified ‘donor’ is almost certainly just the most closely related species currently sequenced. This is especially the case for older HGT events where the same foreign gene appears in more than one species, that is, where horizontal transfer predates the divergence of species. However, we did find a number of recent transfers (present in only a single studied species) that were identified as originating specifically from *Wolbachia*, with one example each in *D. ananassae*, *C. briggsae* and *C. japonica* (GF19976, CBG07424 and Cjp-ubc-6, respectively).

Our method also identified putative HGT from viruses: while rare in both *Drosophila* and *Caenorhabditis*, up to 50 more foreign genes of viral origin per species were identified in the primates (‘Class V’: Additional files 2 and 3). The majority of such genes only align to viral and metazoan sequences, making the direction of transfer unclear, and therefore we excluded them from the rest of our analysis.

### Foreign genes are as likely to contain introns as native genes

The many foreign genes that originate from bacteria would originally have lacked introns, but may have gained them while becoming adapted to the recipient species (domestication). To test this we looked at whether bacterial-origin foreign genes have introns. The *Drosophila* species generally have too few foreign genes to perform the analysis, but in three *Caenorhabditis* species (all except *C. japonica*) and all primates the percentage of bacterial-origin foreign genes with introns is around 95%. For all three classes of foreign gene (C, B and A), there was no significant difference between the proportion of bacterial-origin foreign genes with introns and the proportion of native genes with introns (as measured by a chi-squared test; Additional file 2). The same was true for foreign genes as a whole (all origins; Additional file 2). This observation also makes it unlikely that the detected HGT is actually contamination of the genome with bacterial sequences, as these would lack introns. The exception, *C. japonica*, has significantly fewer bacterial-origin foreign genes with introns than native genes in all three classes (*P* < 8E-6), averaging only 29% of bacterial-origin foreign genes with introns. It also has significantly fewer class A foreign genes with introns than native genes with introns (*P* < 0.001) as discussed below.

### Horizontal gene transfer is both ancient and ongoing

To determine whether the detected HGT is ancient (prior to the divergence of the studied taxon), or has occurred throughout the evolution of a particular taxon, we mapped the foreign ortholog groups (representing founding HGT events) for each taxon onto the corresponding phylogenetic trees. In *Drosophila* species, there is a broad correspondence between length of branch (time) and the number of HGT events along each branch, suggesting that HGT has occurred throughout *Drosophila* evolution and is likely to be ongoing (Figure 1).

The same can be inferred for the *Caenorhabditis* species. Interestingly, a much larger number of HGT events have occurred in *C. japonica* than in the other studied *Caenorhabditis* species or their common ancestors, and its foreign genes also have different properties: it is the only species studied where significantly fewer multi-exon genes are found among foreign genes of prokaryotic origin than among native genes (Additional file 2). Transferred prokaryotic genes presumably require some time to acquire introns, and the lower proportion of intron-containing foreign genes is consistent with comparatively recent HGT events. An alternative explanation is that the *C. japonica* genome is contaminated, since around twice as many of its foreign genes are unlinked to native genes as in other species (Additional file 2). However, even if all unlinked genes are considered to be contamination and are discounted, there would still be more HGT events unique to *C. japonica* than unique to the other studied *Caenorhabditis* species.

The distribution of transfer events is different in the primates, with most foreign groups mapping to the base of the tree (a common ancestor of primates), suggesting that the majority of HGT in primates is ancient. In these cases we are not inferring that the HGT event occurred in the most recent common ancestor of all primates, but that it occurred sometime between the common ancestor of Chordata and the common ancestor of the primates, that is, prior to the time period shown in Figure 1. For example, in the case of HAS1 (Figure 3), which is found in a wide variety of chordates, the HGT event likely occurred soon after the common ancestor of Chordata arose.

### Foreign genes undergo duplication and are distributed throughout the genome

Horizontally acquired genes can undergo duplication and diversification: for example, the three hyaluronan synthases in *Homo sapiens* belong to the same ortholog group and probably result from a single transfer event, followed by duplications. We observed the same scenario for other genes in *H. sapiens* (for example, the four peptidyl arginine deiminases and the nine PRAME family members; Additional file 3), and also in other species. In an extreme case (the O-acyltransferases belonging to the same ortholog group in *C. elegans*; Additional file 3) as many as 30 genes probably derive from a single HGT event.

To ask whether there are ‘hotspots’ undergoing more frequent HGT in the studied genomes, we plotted the locations of foreign genes on the chromosomes or scaffolds of the respective genomes (Additional file 5: Figure S3). We found no evidence for ‘hotspots’, but the limited number of HGT events per species and the frequent occurrence of chromosomal rearrangements during evolution, which complicate cross-species comparisons, make it difficult to draw reliable conclusions.

### HGT is a general feature of chordate genomes

Because there is limited information on HGT in the chordates, we also identified foreign genes for 14 other vertebrate species (Additional file 2). We find 60 to 240 class C genes (approximately 0.4% to 1.3%) across all of these species, in line with our findings for *Drosophila*, *Caenorhabditis* and primates, suggesting that HGT is not restricted to a few animal groups. We did not try to identify class A and B genes, as our method does not produce reliable ortholog groups for species separated by large evolutionary distances.

## Discussion

HGT occurs at low, but appreciable, levels across all the animal species we examined; it has occurred over time and is still occurring; it mainly originates from bacteria and protists; and the genes concerned frequently code for enzyme activities. Interestingly, overall levels of HGT do not appear to be conspicuously different in vertebrates and invertebrates. This is surprising given the difference in complexity between the groups, but may be explained by the observed older HGT in primates, suggesting that the vertebrate HGT may have occurred at an earlier stage of vertebrate evolution. All animal genomes we examined contain expressed foreign genes and therefore unusual circumstances, such as an asexual lifestyle and desiccation tolerance, are not required for transfer, although such characteristics might accelerate HGT rates, as in the bdelloid rotifer.

We have been able to improve on earlier studies of HGT in model organisms [19,30] for two main reasons. The first is the much larger number of species, including many more metazoans, now represented in the sequence databases used for comparison. This reduces the false discovery rate, by increasing the number of species in which a gene must be lost before it is incorrectly called HGT in the species in which it is found. In the original human genome paper [19] only two metazoans were used, *D. melanogaster* and *C. elegans*, while we used hundreds of species. We also examined transfer from multiple taxa, rather than just bacteria [19] or fungi [30], which reduces the false negative rate: one of the rebuttals of the original human paper [20] correctly rejected hyaluronan synthases as prokaryote-to-vertebrate transfer, but failed to identify it as fungus-to-vertebrate transfer because fungal sequences were not considered as potential donors.

The second major improvement is the use of multiple closely related animal species when testing for HGT, allowing the construction of ortholog groups. This reduces the false discovery rate by controlling for spurious alignments that could incorrectly identify a gene as foreign in the minority of species in a group. In particular, this increases our accuracy when searching for older HGT, which is likely to be found in more than one species.

The clearest demonstration of HGT would depend on reliable identification of the donor species, but the source of a foreign gene can only be traced to the most similar extant organism with a sequenced genome. The identification of the donor species for anything but the most recent HGT is further complicated by the sequence evolution of both recipient and donor; as a consequence, absolute certainty in the assignment of most HGT is unachievable. To accommodate this issue, we have defined a set of HGT classes that display differing levels of phylogenetic validation. While class A HGT has the highest degree of validation (88%), the levels of class C HGT are more directly comparable to those of recent studies – because in most cases closely related species are not available, so ortholog groups cannot be constructed – yet even this least stringently selected class of genes is 55% validated (amounting to an additional eight to 58 validated genes per species on top of those in class A).

Although phylogenetic validation is seen as the ‘gold standard’ for HGT discovery, it is important to note that many class A foreign genes (68%) have no metazoan alignments (bitscore <50) so need not be, indeed cannot be, validated as HGT in this way. In these cases, the lack of matches with metazoan genes, together with clear matches to non-metazoan sequences, is sufficient to demonstrate HGT, while phylogenetics can be used to suggest the origin of such sequences. Another issue with phylogenetic validation arises when it is used in an automated manner: for the highest degree of accuracy, trees should only contain orthologs, but determining orthologs from very large sets of sequences requires manual annotation. Phylogenetic trees for HGT validation are also sensitive to contamination, which is widespread in genomes deposited in UniProt (see Additional file 1). We find around 13% of our not validated trees would be validated if a single metazoan protein, grouping within another taxon without other metazoan proteins, were actually non-metazoan contamination, increasing the level of validated HGT proportionally. As many of these sequences likely are contamination it is clear that without very high quality databases phylogenetic approaches lose reliability.

While most studies of HGT look at isolated species, our use of multiple closely related species to define ortholog groups, and thereby class B HGT, reduces the problem of potential contamination by asking that candidate foreign sequences are present in the whole taxon. We only see a modest increase in phylogenetic validation between class C (where ortholog data were not used) and class B HGT (55% to 65%), but this is based on the use of high quality genomes and we would expect a bigger increase when using lower quality genomes.

Our analysis probably underestimates the true extent of HGT in animals for several reasons. First, we set a conservative threshold for the HGT index, that is, *h* ≥ 30, to minimise the false positive rate, but there are probably genes below this threshold that are also horizontally acquired. Second, although hard to detect with available data, metazoan-to-metazoan HGT remains plausible and is known for some host-parasite relationships [36]. Some of these transfers may be mediated by viruses, and in our study we specifically excluded potential virus-mediated HGT due to ambiguity in the direction of transfer. Third, eukaryotic genome projects routinely remove bacterial sequences from their assemblies on the assumption that they are contamination; for instance, this has resulted in the removal of all previously reported HGT from the *D. ananassae* genome. As a result, we may have missed further examples of bacterial HGT in our study, and such screening may explain the lower levels of HGT seen in the *Drosophila* species. While some screening for contamination is clearly necessary, the potential for apparently bacterial sequences to originate from HGT should not be ignored during genome assembly; this observation emphasises the importance of using high quality genome assemblies, as we did here, when searching for HGT.

It is important to consider the likelihood of other explanations for our results. The most obvious is the possibility that the observed foreign genes were inherited by vertical descent, but have been lost from all other observed metazoan species outside the phylum of interest. Increasing the number of metazoan species with high quality genomes and transcriptomes will in future help shed light on this possibility. In the meantime, we observed a striking difference between all classes of HGT and the native genes found in chordates, but not in other metazoans. Thus, genes that are apparently missing in animals other than chordates are significantly less likely to have GO terms for enzyme activities than other native genes (4% vs. 20%), while in contrast the HGT candidates are significantly more likely to have GO terms for enzyme activities (42% vs. 26%). While we cannot completely rule out gene loss as an explanation for our observations, these findings, together with the other lines of evidence presented, suggest that HGT is the more likely explanation.

## Conclusions

Although observed rates of acquisition of horizontally transferred genes in eukaryotes are generally lower than in prokaryotes, it appears that, far from being a rare occurrence, HGT has contributed to the evolution of many, perhaps all, animals and that the process is ongoing in most lineages. Between tens and hundreds of foreign genes are expressed in all the animals we surveyed, including humans. The majority of these genes are concerned with metabolism, suggesting that HGT contributes to biochemical diversification during animal evolution.

## Materials and methods

### Data sources

Genomes, transcriptomes, proteomes and gffs for *Drosophila* species were obtained from FlyBase [37,38]. Additional annotation was obtained from FlyMine [39,40]. All data on *Caenorhabditis* species were obtained from WormBase [41,42], while data on primate and chordate species were obtained from Ensembl [43,44], with the exception of ortholog groups which were obtained from OrthoMCL [45,46]. Genome versions used are shown in Additional file 2.

### Determination of HGT index, *h*

The workflow for this step is shown in Additional file 5: Figure S4. For each studied species, all transcripts were aligned with blastx [47] to two protein databases derived from complete proteomes in UniProt, one consisting of metazoan proteins (excluding proteins from species in the same phylum as the studied species - Arthropoda, Nematoda or Chordata), the other of non-metazoan proteins. The HGT index, *h*, was calculated by subtracting the bitscore of the best metazoan match from that of the best non-metazoan match [12]. The majority of transcripts in all species have *h* <0, indicating they match better to metazoan proteins, as would be expected from vertical gene transfer through the tree of life, where they have had longer to diverge from non-metazoan proteins than from metazoan ones. Therefore, transcripts with *h* >0, which are less diverged from non-metazoan proteins than metazoan, should have been acquired by horizontal transfer from non-metazoans. Rather than just take all transcripts with *h* >0, we require that they align much better to non-metazoan proteins than to metazoan proteins and define candidate (class C) HGT genes as those with *h* ≥30 that also have a best non-metazoan bitscore ≥100. The threshold of 30 was chosen because detailed analysis in our earlier paper [12] found this threshold to be the best trade-off between sensitivity and specificity. As bitscore is a logarithmic measure of sequence similarity, 30 is a large difference in alignment quality. For each gene, *h* was inherited from the transcript with the match with the highest bitscore.

For the *Drosophila*, *Caenorhabditis* and primate species studied, all proteins in each group were aligned to each other with blastp, using a cutoff of 1E-5. Ortholog groups were determined from this alignment using MCL with I = 15 [48]. This value was determined by comparing the ortholog groups to preexisting groups (more details in Additional file 1).

For each class C gene, the average *h* value of the members of its ortholog group was determined (*h*_*orth*_); if this was ≥30 the gene was considered to be a class B gene. Class A genes were defined as a subset of class B genes with no metazoan matches with bitscore ≥100 and no members of their respective ortholog group with metazoan matches with bitscore ≥100. Numbers of each class for each species are shown in Additional file 2.

### Phylogenetic validation

We phylogenetically validated all foreign genes that had any metazoan matches with bitscore ≥50 using a method based on that previously used, producing unrooted trees [12]. We used a strict validation, requiring that the trees showed no evidence that the foreign gene was metazoan. The trees were considered validated if the foreign gene was monophyletic either with a single donor taxon or with multiple potential donor taxa and was not monophyletic with the metazoa. In cases where the foreign genes were monophyletic with both the metazoans and the donor(s) the tree was not validated. We did not require the ‘own-phylum’ taxon (Arthropoda, Chordata, Nematoda) to be monophyletic, as in cases of recent HGT the best matches in this taxon are not orthologs to the foreign gene. For further details see Additional file 1.

All phylogenetic trees containing metazoan matches with bitscore ≥50 are available at [49].

### Manual validation

The 145 human genes classified as HGT were also subjected to manual validation. The transcript with the best blastx bitscore from the previous analysis was blastx-compared to the non-redundant protein sequence (nr) database, excluding Chordata (taxon id: 7711), Vertebrata (taxon id: 7742) or Metazoa (taxon id: 33208) in turn, using the NCBI website [50,51]. The results were manually inspected and the alignments checked for reliability. The same 145 transcripts were also analysed according to published protocols [12]; in summary, sequences were compared (using NCBI-blast-2.2.27+) [47] against the complete proteomes on UniProt. The comparison was done against kingdom-specific databases containing exclusively Metazoa (taxon id: 33208), Eubacteria (taxon id: 2), Archaea (taxon id: 2157), Fungi (taxon id: 4751), plants (taxon id: 3193) and protist (eukaryotes without Metazoa, Fungi and plants) sequences. Bitscores were recorded for the best hit in each taxon and *h* calculated as described. Results were manually analysed to check for agreement with the analysis using the nr database and the automated analysis.

### Genome linkage tests

For each foreign gene, we identified to which contig/scaffold it mapped and determined whether native genes (for which *h* <30) were also found on that contig. If so the horizontally transferred gene was considered to be linked to a native gene. Results are shown in Additional file 2. Discussion of species with lower than average levels of linkage is contained in Additional file 1.

### Functional characterisation of genes

To determine whether horizontally transferred genes encode enzymes, we examined GO annotation [52]. A more direct calculation using EC numbers is not possible due to a lack of EC annotation in most of the species studied. GO terms used in the test species were manually annotated to indicate whether they referred to an enzymatic activity. A hypergeometric test was performed to calculate per species which GO terms were enriched in each class of foreign gene (threshold of *P* ≤0.05). Benjamini-Hochberg multiple testing correction was performed to reduce the false positive rate. We then calculated whether enzymes were significantly over-represented in the enriched versus the non-enriched terms using a chi-squared test (threshold of *P* ≤0.05). Results are shown in Additional file 2.

### Identification of non-HGT genes that are found in chordates and not in non-chordate metazoans

The metazoan species used in the analysis (both the 40 studied and those with complete proteomes in the UniProt database) were placed into a phylogenetic, binary tree based on the NCBI taxonomy (Additional file 5: Figure S5). This tree has six branchpoints between the origin of metazoa and the phyla in which the studied species are found (Arthropoda, Nematoda, Chordata), meaning that a minimum of six gene losses (one at each of these branchpoints) would be required for an HGT event occurring at the base of the phyla to appear to be HGT when in fact it was a result of gene loss. It must also be noted that as not all identified HGTs occur at the base of the phyla, as shown in Figure 1, the number of required gene losses is greater for much of the HGT.

For each studied primate species we identified all non-HGT (that is, native) genes that have been lost at least at these six branchpoints using a BLAST alignment to the metazoan database from UniProt. For each branchpoint where a loss must occur there are a varying number of species; if there were no matches with bitscore ≥100 to any proteins in these species then a gene loss was considered to have occurred in the relevant branch. These non-HGT genes were then analysed based on their GO terms, as done previously for the HGT genes (above), with the comparison made to non-HGT genes that did not have this pattern of loss.

### Introns

To determine whether introns are present at significantly different rates in foreign vs. native genes, we compared the number of native genes with introns to the number of genes of each class of HGT with introns using a chi-squared test (threshold of *p* ≤0.05). Results are shown in Additional file 2.

Validation and discussion of methods is contained in Additional file 1.

### Description of additional data files

The following additional data are available with the online version of this paper. Additional file 1 contains validation and discussion of the methods used in this paper, as well as the legends for the other additional files. Additional file 2 is a table of HGT levels and analyses for all species. Additional file 3 is a table of the horizontally acquired genes in *H. sapiens*, *D. melanogaster* and *C. elegans*, listed by class. Additional file 4 is a table of *H. sapiens* genes previously identified as horizontally transferred. Additional file 5 contains the supplementary figures - Figure S1 shows the phylogenetic trees for the human genes discussed in the section ‘Identification of new foreign genes and confirmation of previously reported examples’. Figure S2 shows the amino-acid alignment between the *C. elegans* trehalose-phosphate synthase gene *tps-1* and the *D. melanogaster* trehalose-phosphate synthase gene Tps1. Figure S3 shows the position of foreign genes on the *D. melanogaster* and *C. elegans* chromosomes. Figure S4 shows the workflow used to identify HGT. Figure S5 shows the simplified phylogenetic tree of species used in analysis. Figure S6 shows the phylogenetic trees for the six human genes originally labelled as horizontally acquired, and later rejected, which are reclaimed.

## Additional files

Additional file 1:
**Validation and discussion of methods.**
Additional file 2:
**HGT levels and analyses for all species.**
Additional file 3:
**Horizontally acquired genes in H. sapiens, D. melanogaster and C. elegans, listed by class and functional classes.**
Additional file 4:
**H. sapiens genes previously identified as horizontally transferred.**
Additional file 5:
**Supplementary figures.**
