# Supplementary material for: Expression of multiple horizontally acquired genes is a hallmark of both vertebrate and invertebrate genomes
Source: Genome Biol. 2015 Mar 13;16(1):50. doi: 10.1186/s13059-015-0607-3 (PMC4358723; doi:10.1186/s13059-015-0607-3)
Supplement: Additional file 1: — Validation and discussion of methods. [file 13059_2015_607_MOESM1_ESM.pdf]

Validation and discussion of methods

Construction of ortholog groups and trees

To determine parameters for MCL [48] our groups were compared to existing groups. For *Drosophila* species, our calculated ortholog groups (16,704 groups) were compared to the homolog groups available on FlyMine (7,871 groups; originally from 52) and we checked for mismatches between the sets. With I = 15, we placed only 4,150 proteins in groups not containing all the orthologs present in FlyMine (3% of total proteins) and removed 239 groups (also 3% of total groups). Sixty of the removed groups were completely merged into other groups, which may represent paralog groups separated in the FlyMine data. A further 138 groups were found split into multiple groups with the only other proteins in these groups coming from a single other group, which may again represent paralog groups separated in the FlyMine data that we are imperfectly separating. This leaves only 41 (0.5% of total) groups in which the situation is more complex. Our groups also contained an additional 45,132 proteins (34% extra) not contained in the FlyMine groups. For *Caenorhabditis* and primate species, we could not find preexisting ortholog groups covering such a large proportion of the proteins. However, we compared our groups to those groups we could find, and found similarly high levels of correspondence. For instance, the *H. sapiens-Pan troglodytes* ortholog groups from release 5 of the OrthoMCL database [46] showed perfect concordance with our groups, with all proteins in the OrthoMCL groups being found with the same proteins in our groups.

These ortholog groups were then mapped to the phylogenetic trees for the three taxa. It was assumed that independent gain of the same ortholog set had not occurred in different lineages, but that loss of an ortholog set was possible. Groups are therefore placed at the node containing all species with proteins in the group, irrespective of whether there are any other species also at

the node. Thus, the original horizontal transfer event that gave rise to each HGT ortholog group probably occurred somewhere along the branch to which it is assigned. To construct the trees, the phylogenetic relationships were derived from different sources according to taxon, i.e. *Drosophila* [52], *Caenorhabditis* [53] or primates [54].

Phylogenetic validation

We phylogenetically validated all foreign genes that had metazoan hits with bitscore ≥ 50. For each gene meeting this criterion, the transcript with the best bitscore from the blastx alignment used for *h* calculation was translated and aligned using ClustalW2 to the five best hits that were in the same translation frame for each of the five donor taxa (archaea, bacteria, fungi, plants, protists) as determined by the same blastx alignment, as well as the five best hits that were in the same translation frame for a blastx alignment to the same phylum as the studied species - Arthropoda, Nematoda or Chordata - giving a maximum of 31 sequences. Each alignment was then trimmed to exclude regions where only one of the sequences was present, and phylogenetic trees were built in PhyML from amino-acid sequences using a JTT model [55]; branch support was calculated with the aLRT (approximate Likelihood-Ratio Test) method. We used a strict validation, requiring that the trees showed no evidence that the foreign gene was metazoan. The trees were considered validated if the foreign gene was monophyletic either with a single donor taxon or with multiple potential donor taxa and was not monophyletic with the metazoa. In cases where the foreign genes were monophyletic with both the metazoans and the donor(s) the tree was not validated. We did not require the 'own- phylum' taxon (Arthropoda, Chordata, Nematoda) to be monophyletic, as in cases of recent HGT the best matches in this taxon are not orthologs to the foreign gene. Results are summarised in Additional files 2 and 3.

During testing to determine the thresholds for HGT we also produced trees (using different thresholds to those used in the main analysis) with 25 sequences in each category for all putative HGT in 14 species (of the 26 used in the main analysis) and found a high degree of concordance, whether validated or not validated, with the corresponding 5-sequences trees (80% of all 5-sequence trees). Of the remaining trees, the majority (another 13% of the original set) were validated in the 25-sequence trees but not in the 5-sequence trees, i.e. the 25-sequence trees suggest that the HGT is real, while the 5-sequence trees suggest that the HGT is false (false negatives). This leaves only a small proportion of the trees (7%) where the 5-sequences trees suggest that the HGT is real, while the 25-sequence trees suggest that the HGT is false (false positives).

Based on our analysis, a number of proteomes in UniProt are markedly contaminated with sequences from organisms outside their own taxa, causing problems for the analysis; consequently, hits from these proteomes were not counted when determining monophyly. The following proteomes were excluded: *Populus trichocarpa* (Western balsam poplar; taxon id: 3694), *Amphimedon queenslandica* (sponge; taxon id: 400682), *Branchiostoma floridae* (Florida lancelet; taxon id: 7739), *Crassostrea gigas* (Pacific oyster; taxon id: 29159), *Nematostella vectensis* (starlet sea anemone; taxon id: 45351), *Oikopleura dioica* (tunicate; taxon id: 34765), *Siniperca chuatsi* (Chinese perch; taxon id: 119488), *Strongylocentrotus purpuratus* (purple sea urchin; *taxon id: 7668), Trichoplax adhaerens* (placozoan; taxon id: 10228). These are by no means the only contaminated proteomes. Looking at all cases where a single metazoan protein is found grouped with another taxon (possible contamination) there are 133 not validated trees (13% of all not validated trees) where this may have caused us to fail to validate the HGT. Some of these proteins might instead themselves be a result of HGT (which would also mean the tree

should be validated). Subsequent to our analysis UniProt has removed many of the sequences that we considered to be contamination.

A further issue with phylogenetic validation is long branch attraction. This affects the results by either misplacing metazoan hits, causing the tree to be incorrectly not validated, or misplacing the foreign gene (and sequences from its own phyla), causing the tree to be incorrectly validated. There are likely cases of the former included among the not validated trees discussed above, especially in trees for the class A foreign genes where the best metazoan hits have a bitscore < 50.

For figure 3, the top five metazoan hits that were not nematodes were added to demonstrate that all metazoans group in the same position in the tree.

Genome linkage tests

For most species, the majority of foreign genes are linked (i.e. they are present in DNA contigs adjacent to native genes), but for *Tarsius syrichta* around 75% of genes are unlinked, suggesting possible contamination. However, we believe this reflects the low quality of the genome assembly, rather than actual contamination, as the levels of HGT detected are comparable to related species and the ortholog grouping shows no HGT events unique to the species that might indicate contamination in that genome (Figure 1). We also see high levels of unlinked genes (around 20%) in *Microcebus murinus* and *C. japonica*. For *M. murinus*, the same arguments as for *T. syrichta* remain true, but *C. japonica* shows higher levels of HGT than other *Caenorhabditis* species and has a large number of unique events. As discussed in the main text this may mean that some of its foreign genes are actually contamination; however, even in the worst case, where all unlinked genes are contamination, this would only reduce levels of HGT to

that of the other *Caenorhabditis* species. *C. japonica* also has higher numbers of HGT events occurring on its branch than the other *Caenorhabditis* species. If all unlinked genes were contamination this would still be the case.

Comparison with previously identified HGT

We compared the foreign genes we identified in *H. sapiens* with the available information [19-22] by linking previously described foreign genes to the current Ensembl gene identifier. Additional file 4 presents the list of previously identified foreign genes in *H. sapiens* combined, where possible, with the current identifiers, and their classification as "horizontally transferred" or metazoan in each paper. Additional file 4 also contains the additional foreign genes we discovered that have not previously been described. In some cases, multiple identifiers in previous papers now correspond to one Ensembl identifier and the original genes had different classifications, so they have been summarised in our table as "ambiguous". For example, in the original human genome sequencing paper, the gene BAA91937 was included in table S24-A as one of the 113 genes deriving from a horizontal transfer event, while the gene gi8922871 was included in table S24-B as one of the 110 genes "initially considered to be candidates for horizontal gene transfer, but later determined not to be" [19]. These two identifiers today correspond to a unique Ensembl gene, ENSG00000152463, so they have been labelled as "ambiguous" in Additional file 4.

At the end of Additional file 4, we summarise the classification of each gene as "discovered" (genes which are foreign according to our analysis and have not previously been described as HGT), "confirmed and re-claimed" (genes previously described as horizontally transferred, and in some cases later rejected, but which we confirm as foreign), "rejected" (genes

previously claimed as horizontally transferred, which we do not find to be foreign, or genes at some point rejected as horizontally transferred that we confirm as native), “deleted” (genes that have been removed from the current public databases) and “?” (genes for which we have been unable to find a correspondence in the current databases). Of the 235 foreign genes previously described, we cannot identify 119 genes and five have been deleted from current databases; of the remaining 111, we reject (in some cases confirming a previously reported rejection) 94 genes, and reclaim or confirm 17 genes. We also discover 128 new genes never previously described as horizontally transferred.

We reclaim six genes originally labelled as horizontally acquired, but later rejected: a hyaluronan synthase (ENSG00000105509, HAS1; Figure 3 and figure S6A in Additional file 5) from fungi, a taxon not considered in previous papers [19,20]; a cytochrome P450 (ENSG00000095596, CYP26A1; Figure S6B in Additional file 5) and an enoyl-CoA dehydrogenase (ENSG00000113790, EHHADH; Figure S6C in Additional file 5), which had been claimed as foreign, but not confirmed by PCR in the original paper [19]; a ribosomal modification protein rimK-like family member (ENSG00000166532, RIMKLB; Figure S6D in Additional file 5); carnosine synthase (ENSG00000172508, CARSN1; Figure S6E in Additional file 5); and an acyl-CoA synthetase family member (ENSG00000183549, ACSM5; Figure S6F in Additional file 5). These examples were previously rejected after phylogenetic analysis [21], but our own phylogenetic analysis shows they are not, in fact, metazoan. In the case of ACMS5 (Figure S6F in Additional file 5), the Chordata sequences (and ACMS5), shown in red in the tree, cluster with two sequences from echinoderms (*S. purpuratus*) and with two sequences from basal metazoans (*N. vectensis*, a sea anemone), which are species whose proteomes we consider to be contaminated and therefore ignore (see “Phylogenetic validation” above), while the only

other metazoan hit is a nematode (*C. remanei*), which is found elsewhere in the tree. The same argument applies to three of the other genes: trees for the cytochrome P450 gene (CYP26A1; Figure S6B in Additional file 5) and the enoyl-CoA dehydrogenase (EHHADH; Figure S6C in Additional file 5) show metazoan sequences divided into two clusters with contaminated proteomes clustering with the chordate proteins (and human gene) and bacteria, far away from the other metazoans. Recalculating the phylogenetic trees after excluding the contaminated proteomes gives essentially the same result, which confirms the foreign nature of the analysed genes (Figure S6 in Additional file 5). The other two genes lack metazoan hits: the ribosomal modification protein rimK-like family member (RIMKLB; Figure S6D in Additional file 5) does not have any significant hit with metazoans, while the carnosine synthase (CARSN1; Figure S6E in Additional file 5) has no significant hits with metazoans in the correct reading frame. Since this analysis a number of sequences in these trees (Figure S6 in Additional file 5, asterisked proteins), which are from proteomes we consider to be contaminated, have been removed from UniProt.

References

52. Drosophila 12 Genomes Consortium, Clark AG, Eisen MB, Smith DR, Bergman CM, Oliver B, Markow TA, Kaufman TC, Kellis M, Gelbart W: **Evolution of genes and genomes on the *Drosophila* phylogeny.** *Nature* 2007, **450:**203-218.
53. Kiontke KC, Felix MA, Ailion M, Rockman MV, Braendle C, Penigault JB, Fitch DH: **A phylogeny and molecular barcodes for *Caenorhabditis*, with numerous new species from rotting fruits.** *BMC Evol Biol* 2011, **11:**339.
54. Perelman P, Johnson WE, Roos C, Seuanez HN, Horvath JE, Moreira MA, Kessing B, Pontius J, Roelke M, Rumpler Y: **A molecular phylogeny of living primates.** *PLoS Genet* 2011, **7(3):**e1001342.
55. Jones DT, Taylor WR, Thornton JM: **The rapid generation of mutation data matrices from protein sequences.** *Comput Appl Biosci* 1992, **8(3)**:275-282.
56. Kearse M, Moir R, Wilson A, Stones-Havas S, Cheung M, Sturrock S, Buxton S, Cooper A, Markowitz S, Duran C, Thierer T, Ashton B, Mentjies P, Drummond A: **Geneious Basic: an integrated and extendable desktop software platform for the organization and analysis of sequence data.** *Bioinformatics* 2012, **28(12)**:1647-1649.

Additional file legends

Additional file 2. HGT levels and analyses for all species. The table shows details of the genome and transcriptome used, levels of HGT in each class, genome linkage analysis and GO enrichment analysis for each class, phylogenetic validation for each class and for genes with $15 \leq h < 30$, taxon matching between the blastx and phylogenetic analysis for class C, and proportions of intron-containing genes in each class. Significance levels in bold are ≤ 0.05; those also highlighted are significantly different in the opposite direction to those not highlighted. Numbers are colour-coded according to HGT class, using the colour scheme as figure 2 (class A: red, class B: orange, class C: blue).

Additional file 3. Horizontally acquired genes in *H. sapiens, D. melanogaster* and *C. elegans,* listed by class. For each species (divided by tab), the columns show gene identifiers, gene name or description, EC number and name, GO identifier and name, transcript identifier, *h* index, likely taxon of origin as calculated from the blastx, ortholog group number and its average *h* index, HGT classification (class A, B or C, with the same colour scheme as figure 2 and Additional file 2), accession number, E-value and bitscore for the best hit against metazoa, archaea, bacteria, fungi, protists and plants in UniProt. The total count of genes in classes A, B and C is also shown for each species. The HGT class assigned is the most stringent applicable. Gene identifiers highlighted in yellow were previously identified by Parkinson and Blaxter [30]. For *H. sapiens* and *D. melanogaster* putative viral HGT (class V HGT) was identified (in separate tabs; no genes were identified as of viral origin in *C. elegans*). HGT from viruses is rare in *Drosophila*, but up to a further 50 candidate HGT genes of viral origin per species were

identified in the primates.

Additional file 4. *H. sapiens* genes previously identified as horizontally transferred. For each gene, the identifiers in previous papers have been matched, where possible, to the Ensembl gene identifier. Whether a specific gene is considered to be horizontally transferred in each of the four main papers [19-22] is indicated, together with the classification from the present analysis. Genes from the original human genome sequencing paper [19] are labelled "HGT confirmed by PCR" or "HGT not confirmed by PCR" according to the data in the paper, or "ambiguous " according to the explanation in Methods. In the following columns, genes are labelled "confirmed" where there is correspondence of identifiers and the gene has been confirmed as foreign, "ambiguous" where either the correspondence is not unequivocal or the gene is not clearly identified as foreign in previous papers, "phylo support" when the gene has been confirmed as foreign by phylogenetic analysis, "phylo rejection" when the gene has not been confirmed as foreign by phylogenetic analysis, "other rejection" when the gene has been not confirmed as foreign based on another type of analysis. A summary of the total number of genes confirmed or re-claimed, discovered, rejected, deleted from databases or unavailable is presented at the end of the table. (See Additional file 1 text above for more information).

Additional file 5 - Supplementary Figure Legends

Figure S1. Phylogenetic trees for the human genes discussed in the main text. For each gene, the tree shows the UniProt accession number of each aligned sequence. The colour scheme is as in figures 3 and 4: the human gene under analysis is shown in orange, while proteins from

chordates are in red, metazoa in black, fungi in pink, plants in green, protists in grey, archaea in light blue and bacteria in dark blue. These trees are unrooted, as seen in figure 3. Numbers indicate aLRT support values for each branch.

Figure S2. Alignment of the *D. melanogaster* and *C. elegans* trehalose-phosphate synthase genes. The amino acid alignment of Tps1 and *tps-1* using Geneious [56]. Green bars indicate exact matches between the sequences.

Figure S3. The position of foreign genes on the *D. melanogaster* and *C. elegans* chromosomes. The positions of all genes on *D. melanogaster* (*A*) and *C. elegans* (*B*) chromosomes is shown in black and the positions of class C foreign genes is shown in red. No foreign genes were found on *D. melanogaster* chromosome 4. Similar patterns are seen across all species and classes.

Figure S4. Workflow to identify HGT.

Figure S5. Simplified phylogenetic tree of species used in analysis. The species used in the analysis were placed in a binary tree based on the NCBI taxonomy. There are six branchpoints between the origin of metazoa and each of the studied taxons (Chordates, Nematodes, Arthropods). Numbers in brackets are NCBI taxonomic identifiers.

Figure S6. Phylogenetic trees for the six human genes originally labelled as horizontally acquired, and later rejected, which are reclaimed. For each gene, the left-hand panel shows

the tree together with the UniProt accession number of each sequence, while the right-hand panel shows the same tree calculated with the exclusion of contaminated genomes (see phylogenetic validation above). The colour scheme is as in figures 3 and 4: the human gene under analysis is shown in orange, while proteins from chordates are in red, metazoa in black, fungi in pink, plants in green, protists in grey, archaea in light blue and bacteria in dark blue. These trees are unrooted, as seen in figure 3 (which shows Figure S1A in Additional file 5). Numbers indicate aLRT support values for each branch. Asterisks indicate sequences that have been removed from UniProt during since the initial analysis.
