# Supplementary material for: Expression of multiple horizontally acquired genes is a hallmark of both vertebrate and invertebrate genomes
Source: Genome Biol. 2015 Mar 13;16(1):50. doi: 10.1186/s13059-015-0607-3 (PMC4358723; doi:10.1186/s13059-015-0607-3)
Supplement: Additional file 5: — Supplementary figures. [file 13059_2015_607_MOESM5_ESM.pdf]

Figure S1A. FTO, fat mass and obesity associated gene (ENSG00000140718)

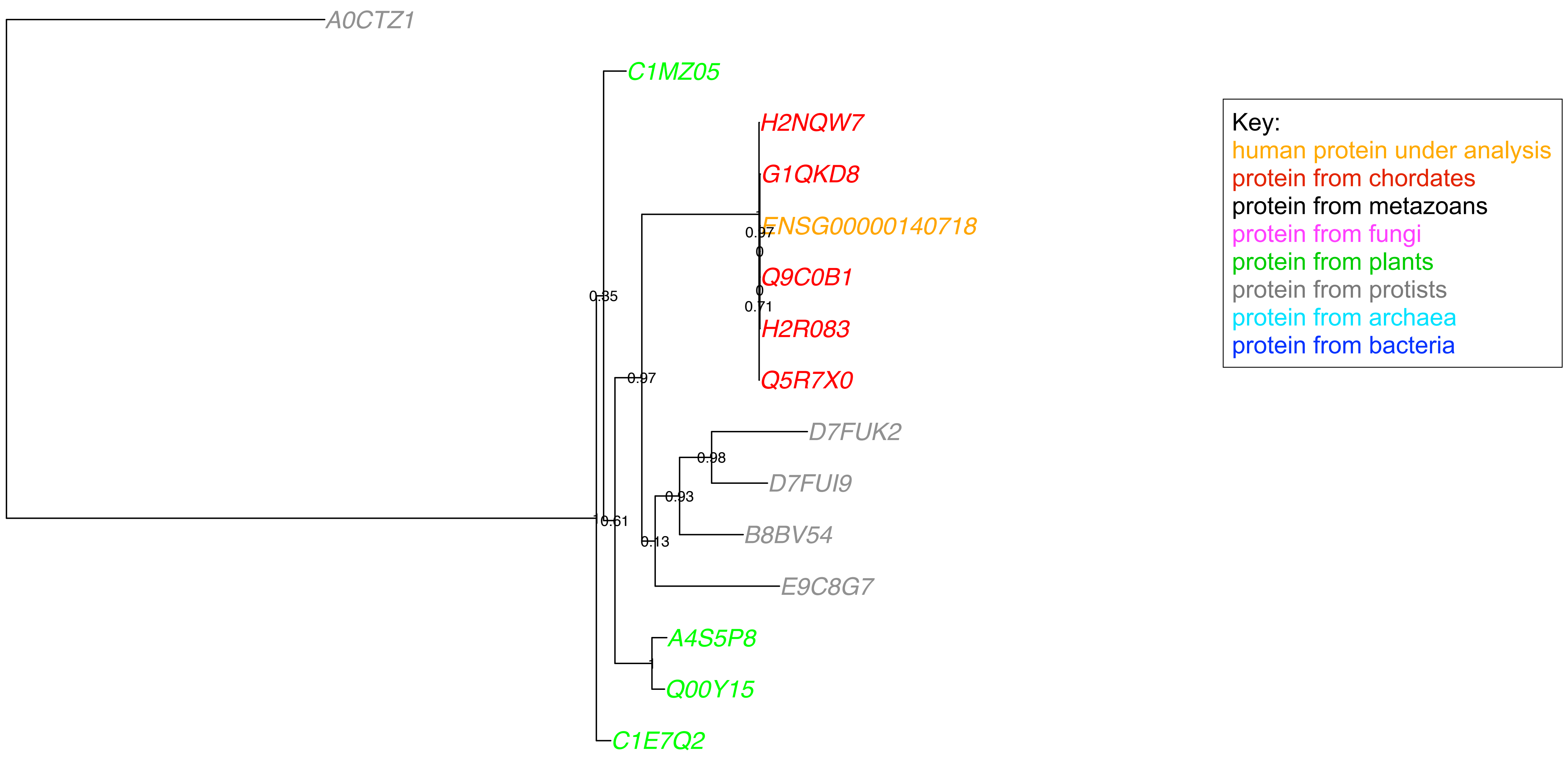

Figure S1B. ABO, transferase A, alpha 1-3-N-acetylgalactosaminyltransferase; transferase B, alpha 1-3-galactosyltransferase (ENSG00000256062)

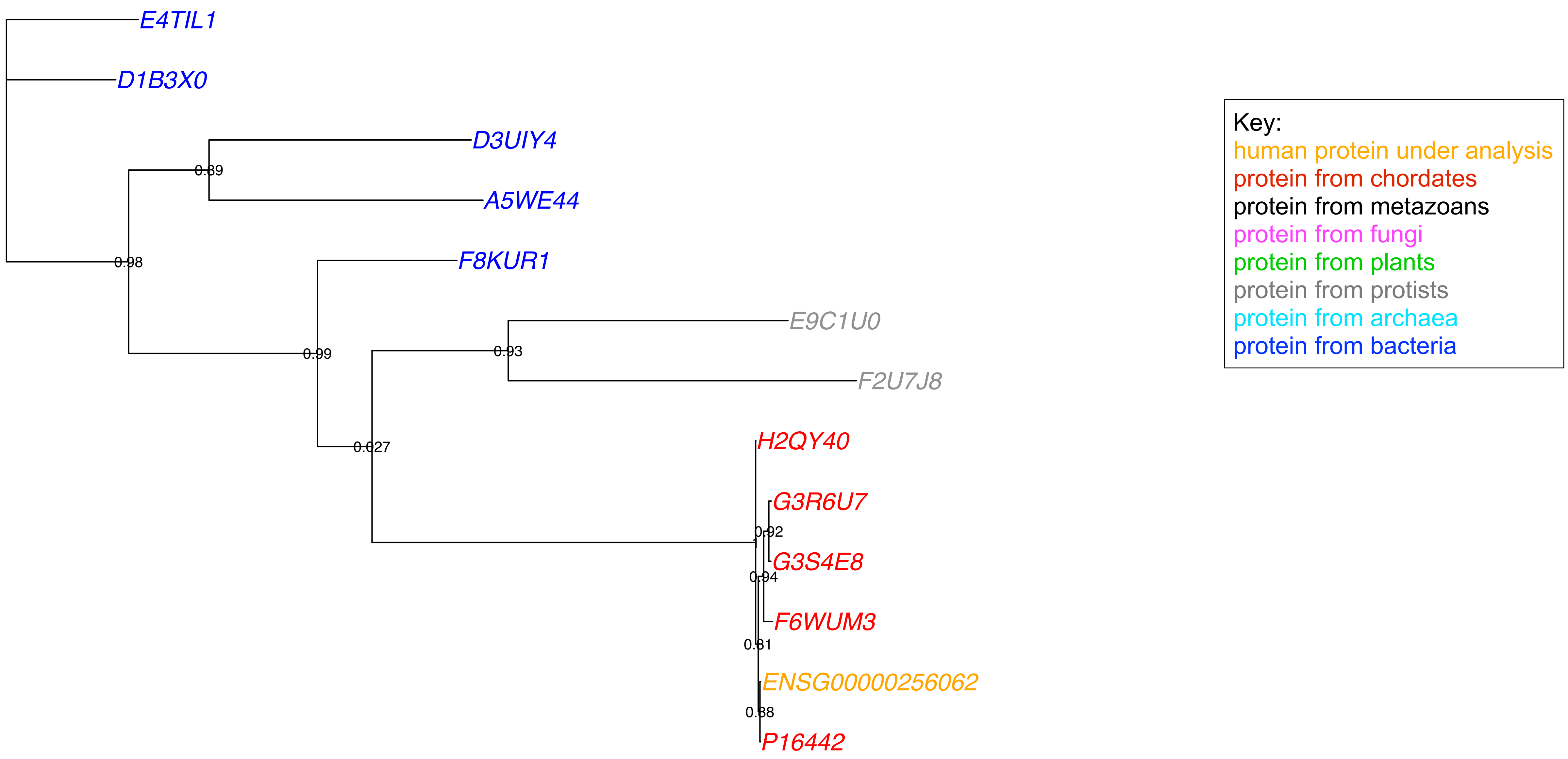

Figure S2.

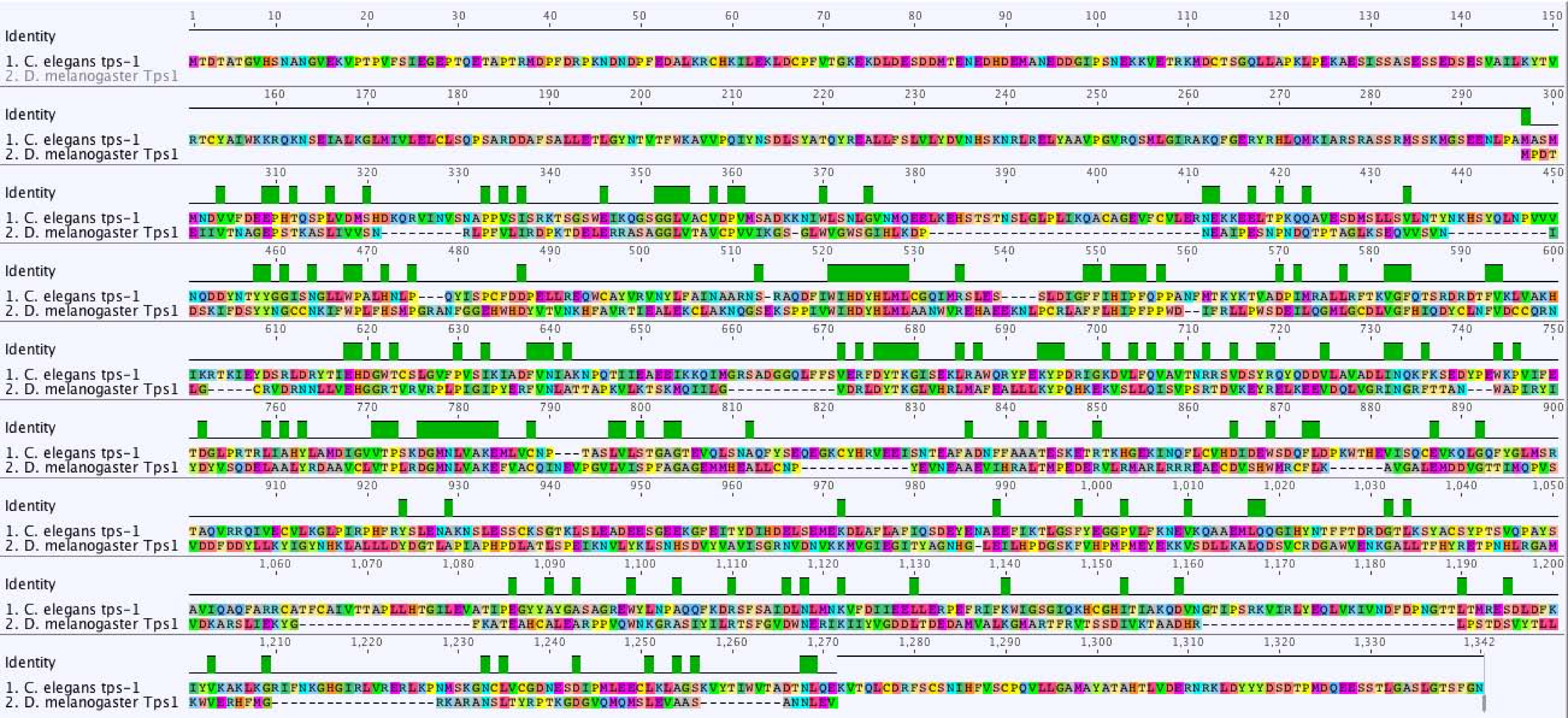

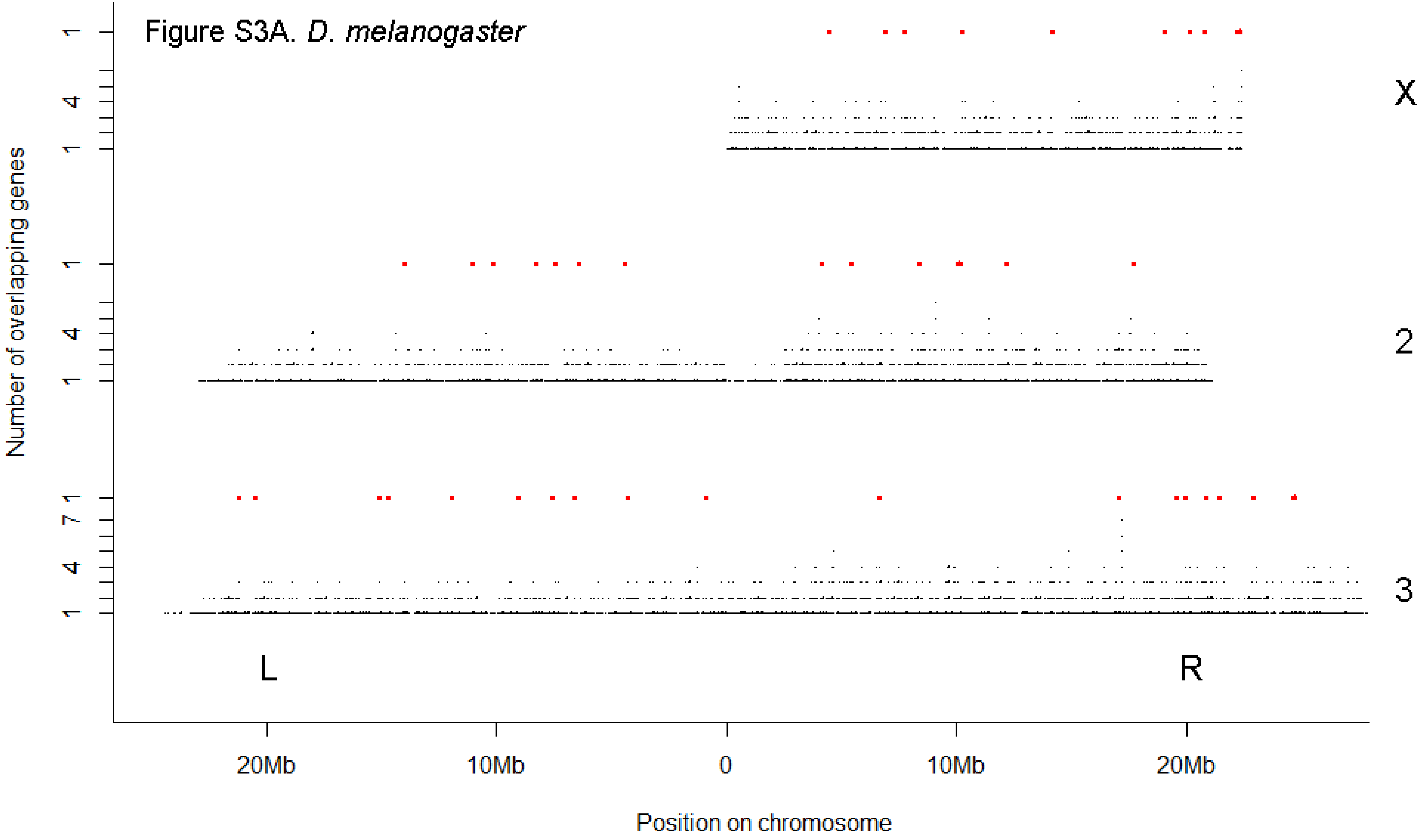

Figure S3A. *D. melanogaster*

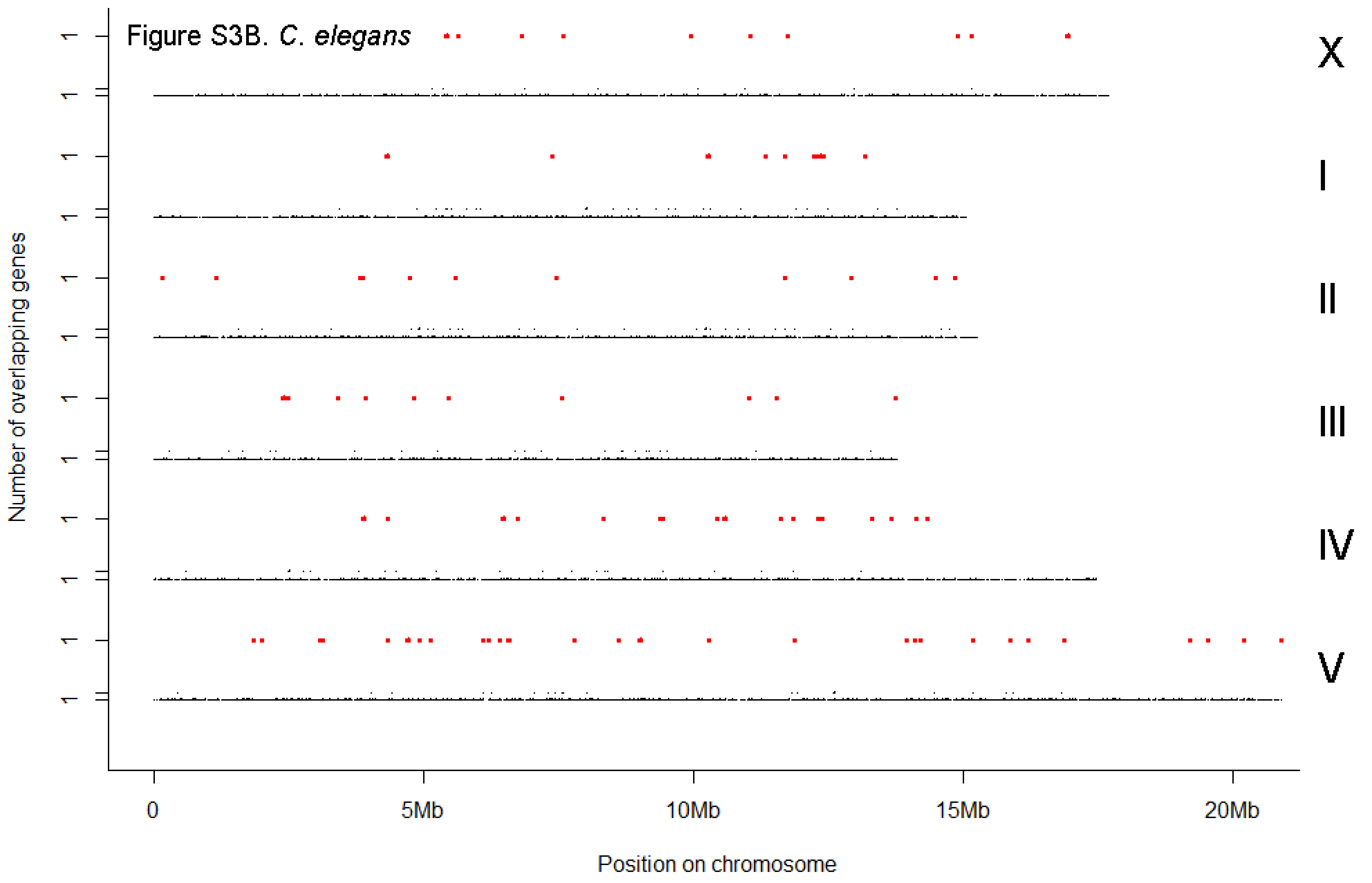

Figure S3B. *C. elegans*
Number of overlapping genes
Position on chromosome
0
5Mb
10Mb
15Mb
20Mb
X
I
II
III
IV
V

Figure S4.

download transcriptome for each species

blastX of each transcript against two databases: Metazoan (excluding phylum under analysis: Arthropoda, Nematoda or Chordata) and Non-Metazoan, record bitscore for each top hit

Calculate *h* (bitscore of best non-metazoan hit - bitscore of best metazoan hit)

if *h* ≥ 30 and best non-metazoan bitscore ≥ 100: class C genes

if *h* < 30, or if *h* ≥ 30 but best non-metazoan bitscore < 100: native genes

construct ortholog sets for the three major taxa (*Drosophila, Caenorhabditis,* primates)

blastp align all proteins in each group to each other (cutoff 1E-5); use these alignments (using MCL) to determine ortholog groups

calculate the average *h* value of the members of each ortholog group (h_{orth})

if *h* >= 30, best non-metazoan bitscore ≥ 100, and h_{orth} >= 30: class B genes

if bitscore of best match to a metazoan < 100 and ortholog groups contain no genes with metazoan matches of bitscore ≥ 100

if *h* >= 30, best non-metazoan bitscore ≥ 100, h_{orth} >= 30, best metazoan bitscore < 100 and no members of the ortholog group has metazoan matches with bitscore ≥ 100: class A genes

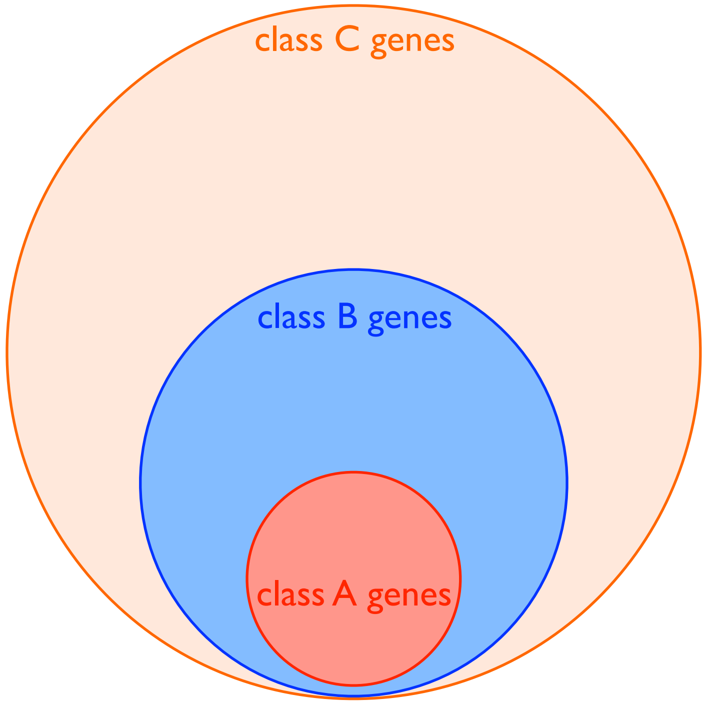

Figure S5.

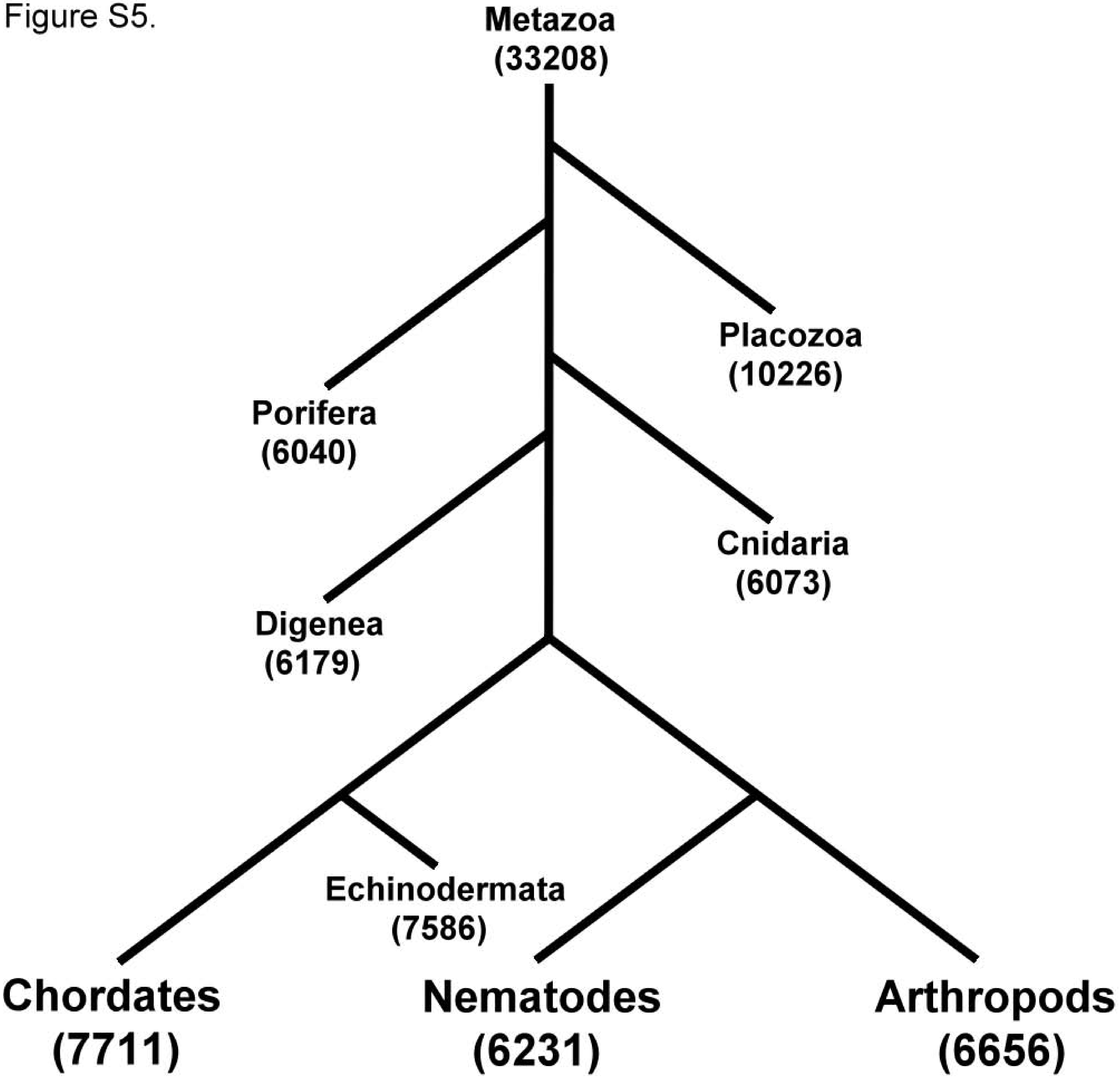
Metazoa
(33208)
Placozoa
(10226)
Porifera
(6040)
Cnidaria
(6073)
Digenea
(6179)
Echinodermata
(7586)
Chordates
(7711)
Nematodes
(6231)
Arthropods
(6656)

Figure S6A. HAS1, hyaluronan synthase 1 (ENSG00000105509)

Key:
human protein under analysis
protein from chordates
protein from metazoans
protein from fungi
protein from plants
protein from protists
protein from archaea
protein from bacteria

* removed from UniProt since analyses

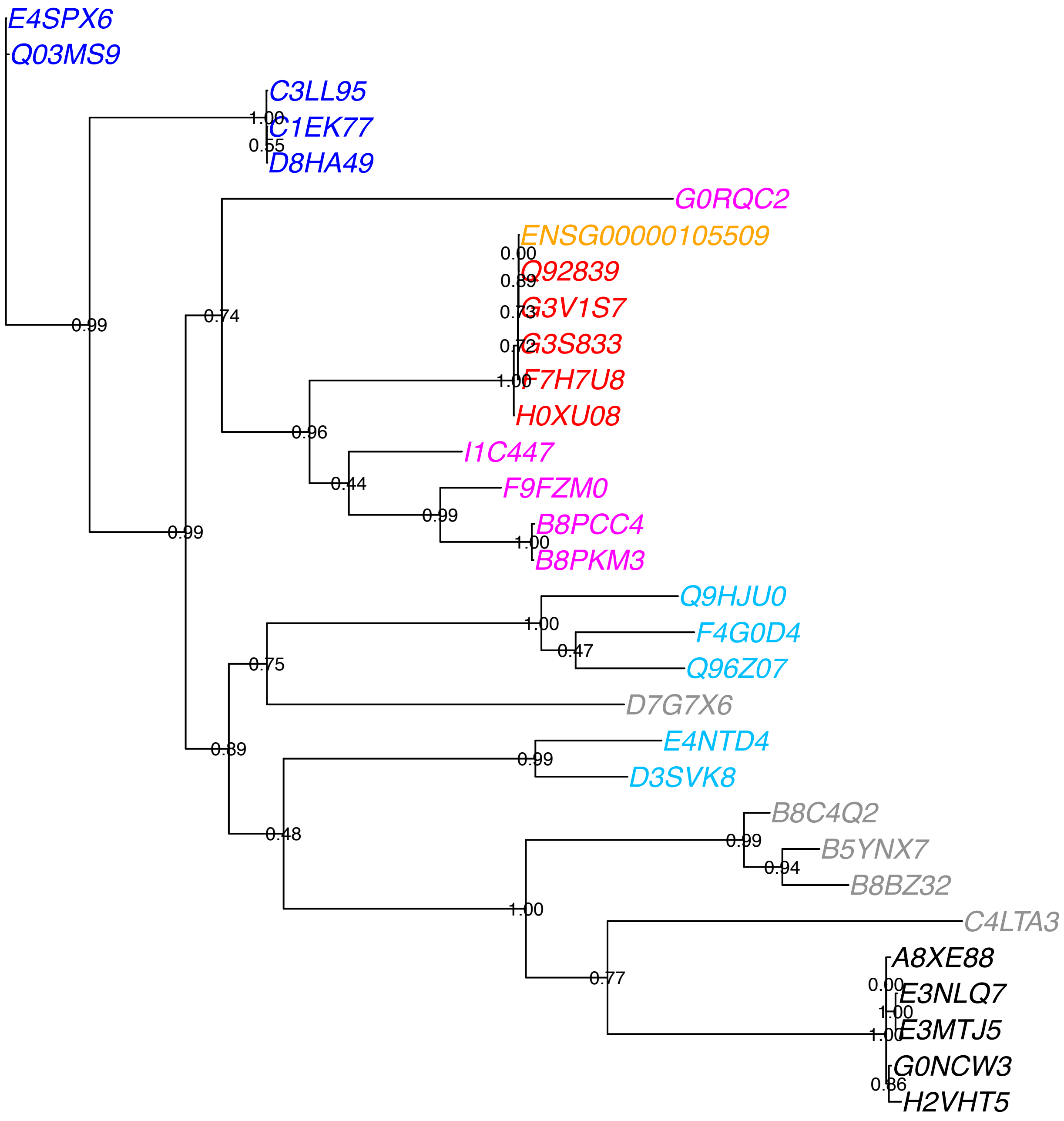

no hits from contaminated genomes

Phylogenetic tree including the contaminated genomes

Phylogenetic tree excluding the contaminated genomes

Figure S6B. CYP26A1, cytochrome P450, family 26, subfamily A, polypeptide 1 (ENSG00000095596)

Key:
human protein under analysis
protein from chordates
protein from metazoans
protein from fungi
protein from plants
protein from protists
protein from archaea
protein from bacteria

* removed from UniProt since analyses

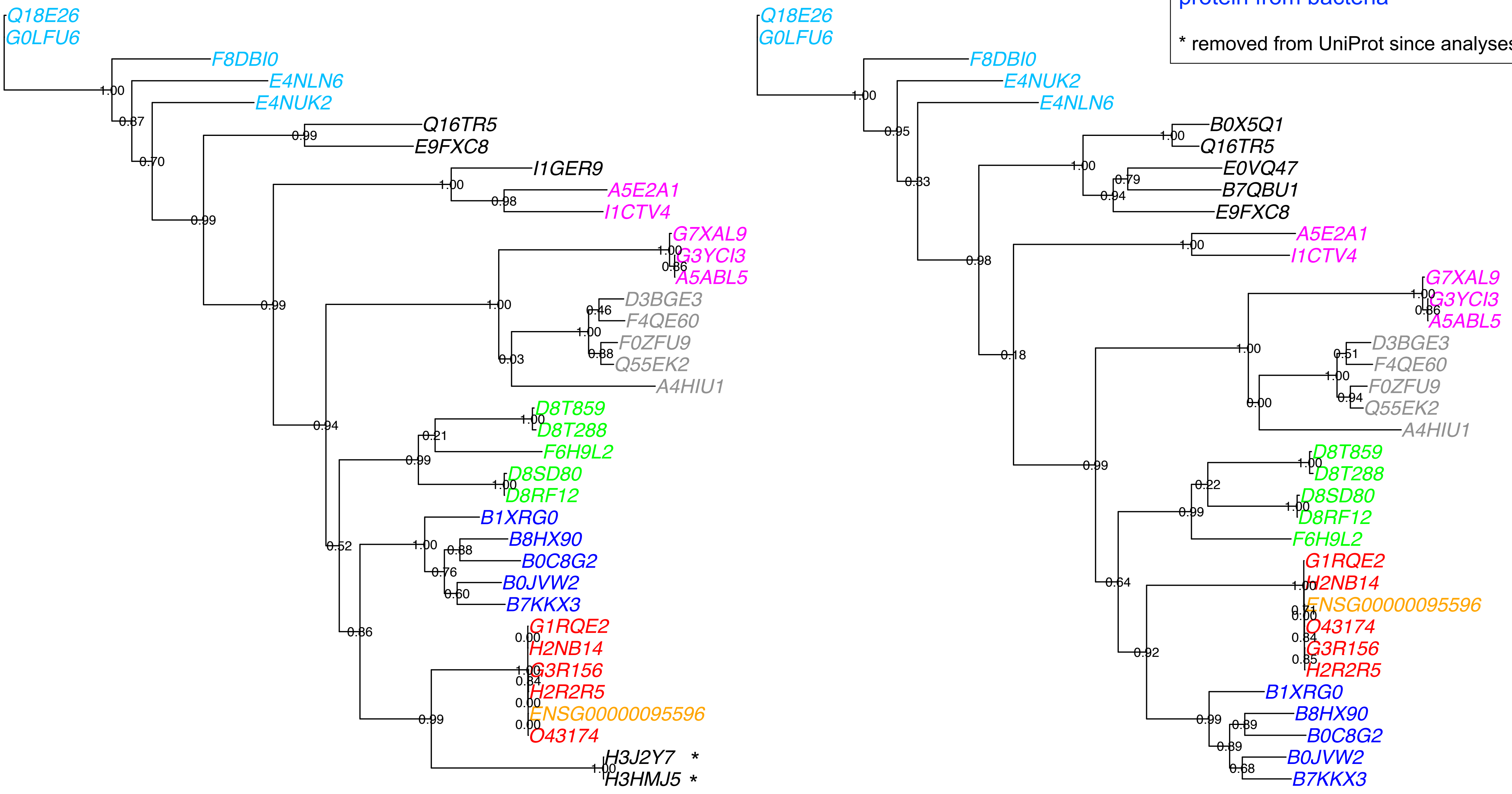

Phylogenetic tree including the contaminated genomes

Phylogenetic tree excluding the contaminated genomes

Figure S6C. EHHADH, enoyl-CoA, hydratase/3-hydroxyacyl CoA dehydrogenase (ENSG00000113790)

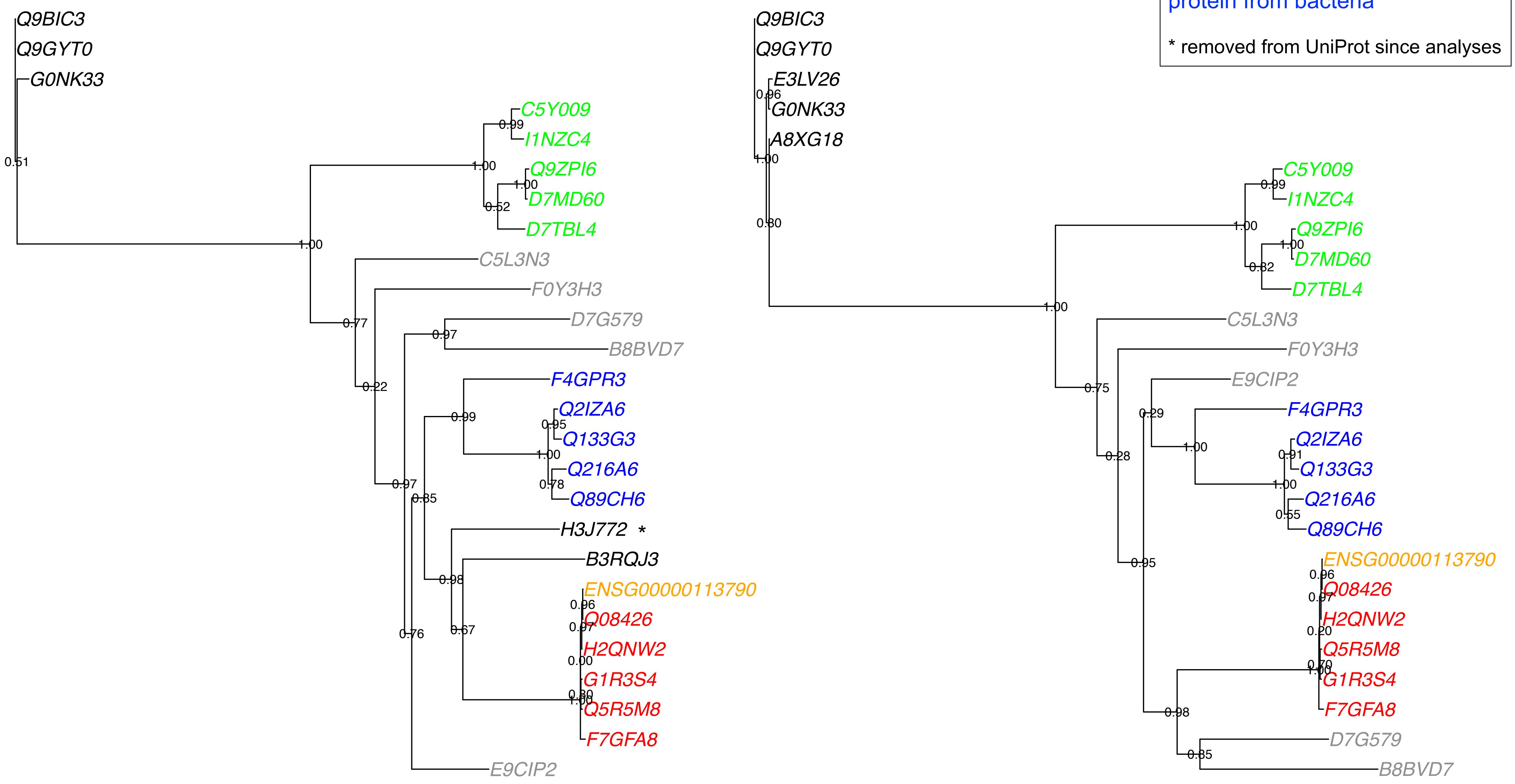

Phylogenetic tree including the contaminated genomes

Phylogenetic tree excluding the contaminated genomes

Figure S6D. RIMKLB, ribosomal modification protein rimK-like family member B (ENSG00000166532)

Key:
human protein under analysis
protein from chordates
protein from metazoans
protein from fungi
protein from plants
protein from protists
protein from archaea
protein from bacteria

* removed from UniProt since analyses

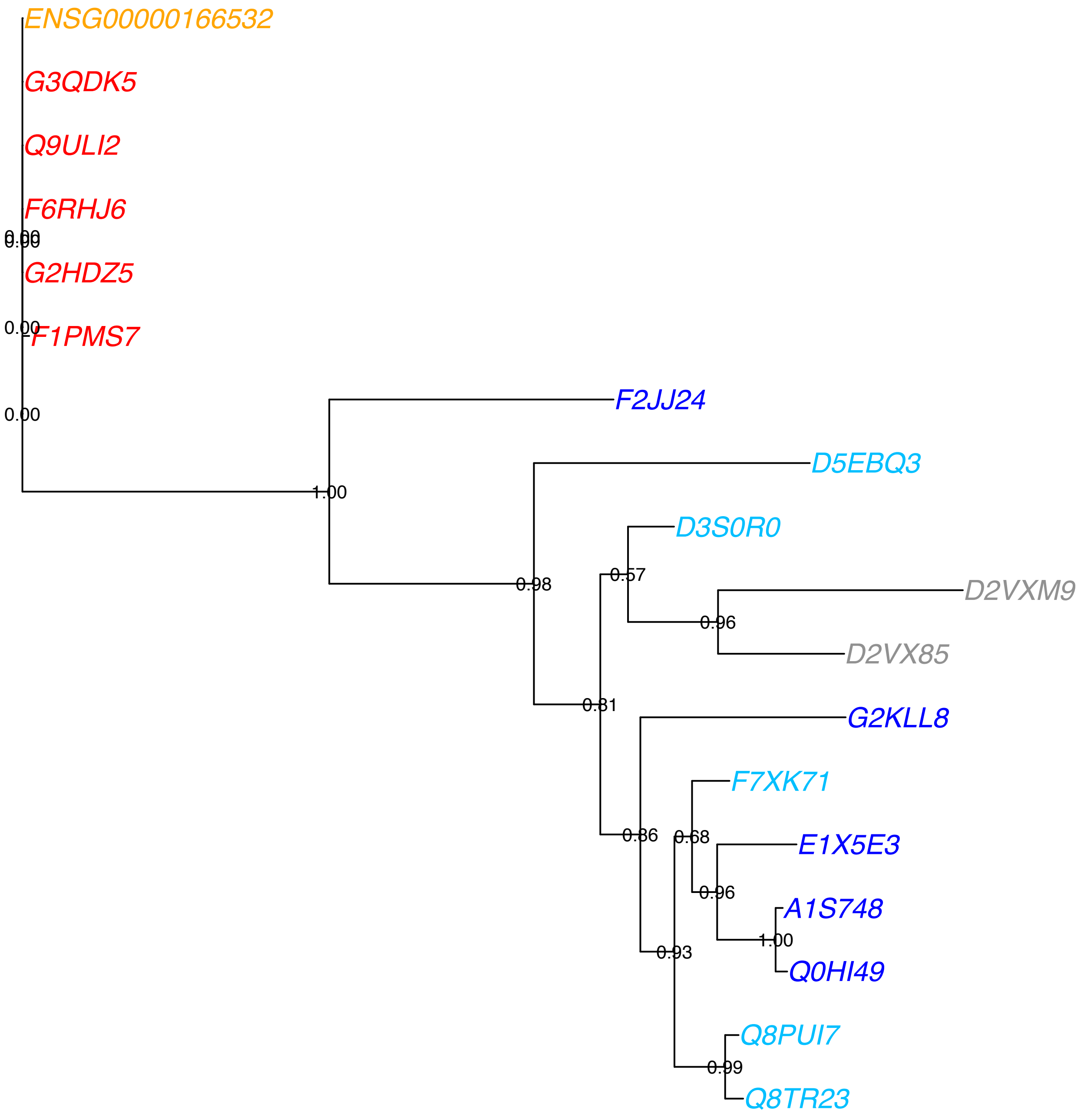

no hits from contaminated genomes

Phylogenetic tree including the contaminated genomes

Phylogenetic tree excluding the contaminated genomes

Figure S6E. CARSN1, carnosine synthase 1 (ENSG00000172508)

Key:
human protein under analysis
protein from chordates
protein from metazoans
protein from fungi
protein from plants
protein from protists
protein from archaea
protein from bacteria

* removed from UniProt since analyses

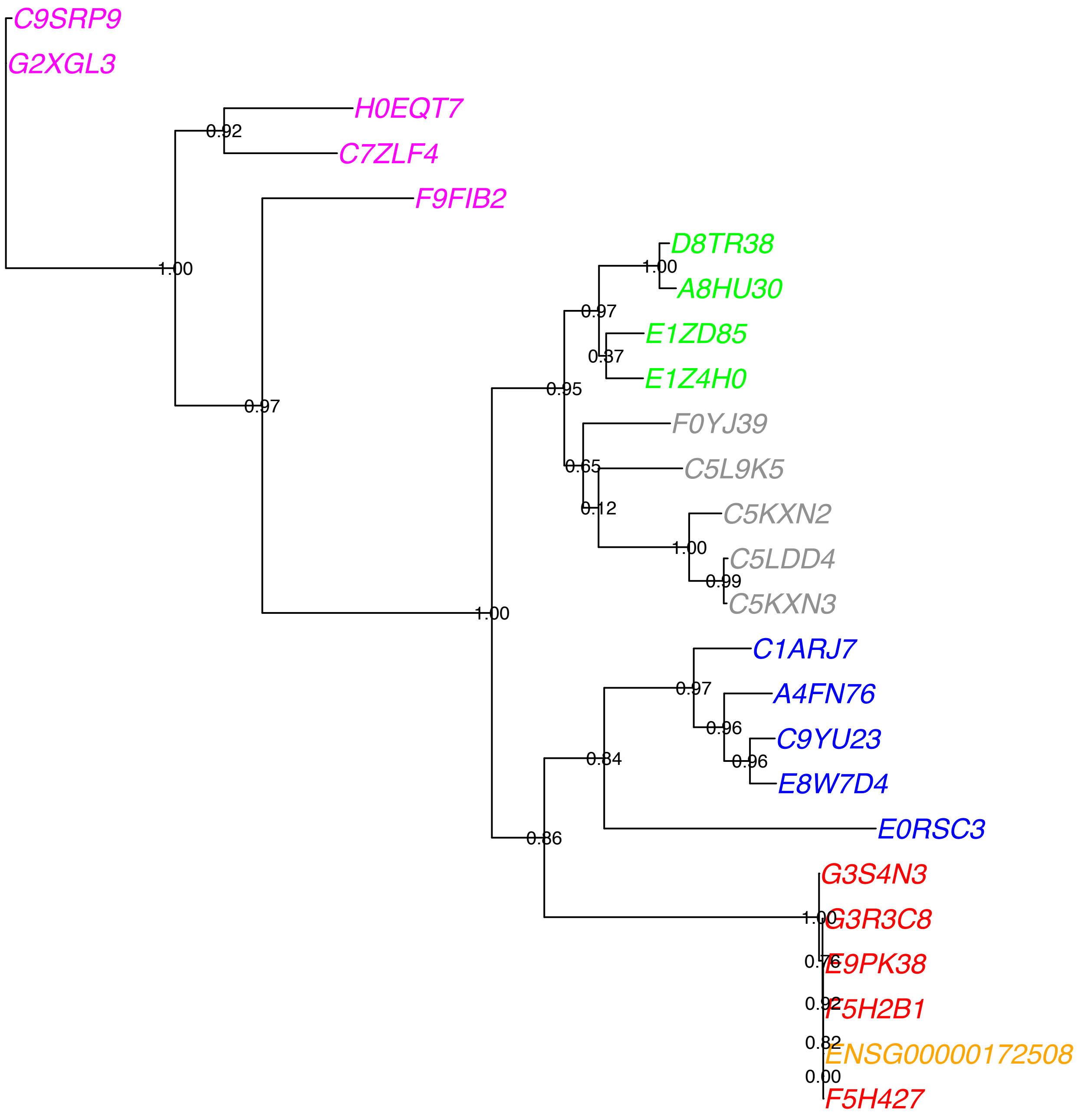

no hits from contaminated genomes

Phylogenetic tree including the contaminated genomes

Phylogenetic tree excluding the contaminated genomes

Figure S6F. ACSM5, acyl-CoA synthetase medium-chain family member 5 (ENSG00000183549)

Key:
human protein under analysis
protein from chordates
protein from metazoans
protein from fungi
protein from plants
protein from protists
protein from archaea
protein from bacteria

* removed from UniProt since analyses

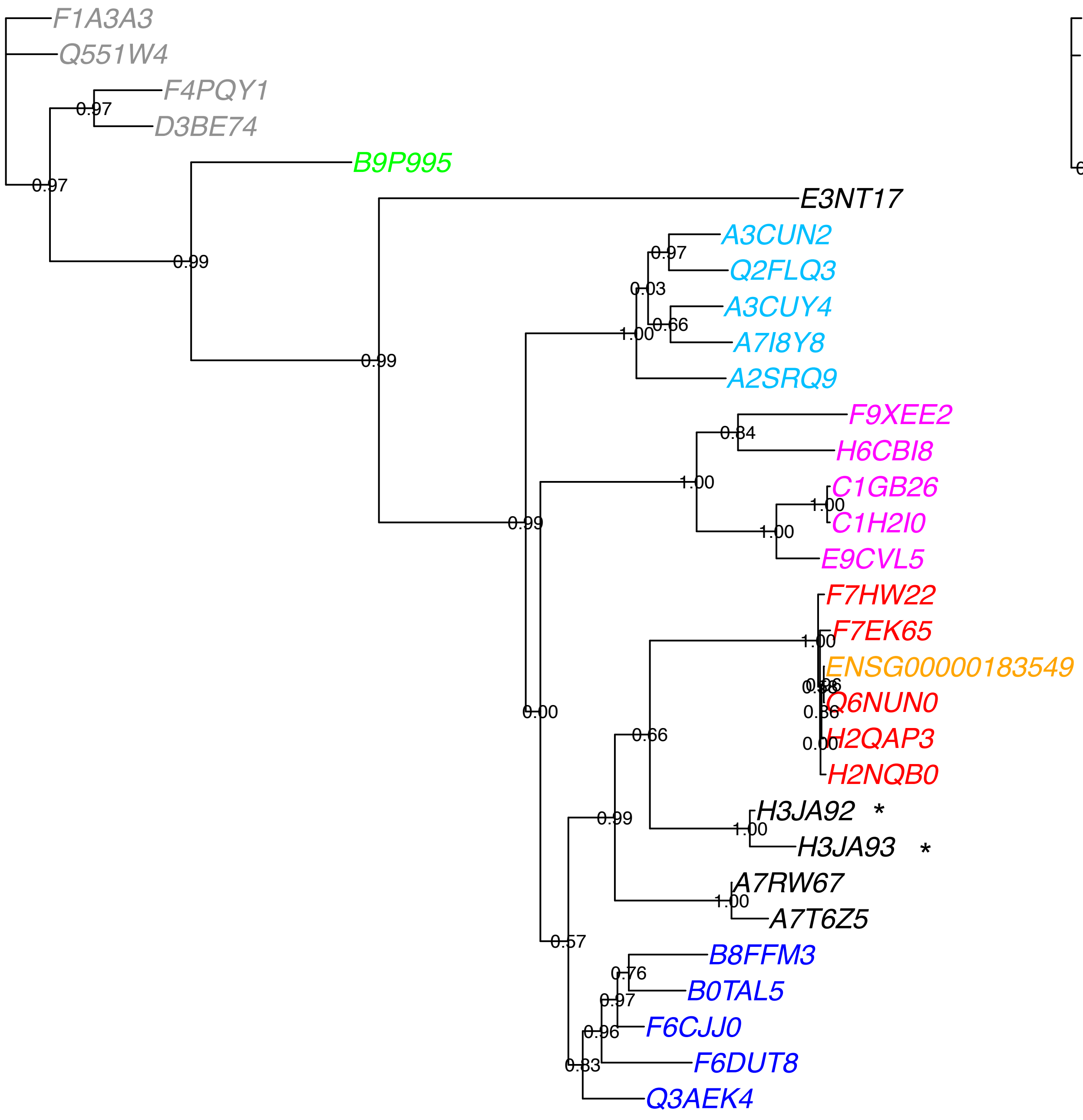

Phylogenetic tree including the contaminated genomes

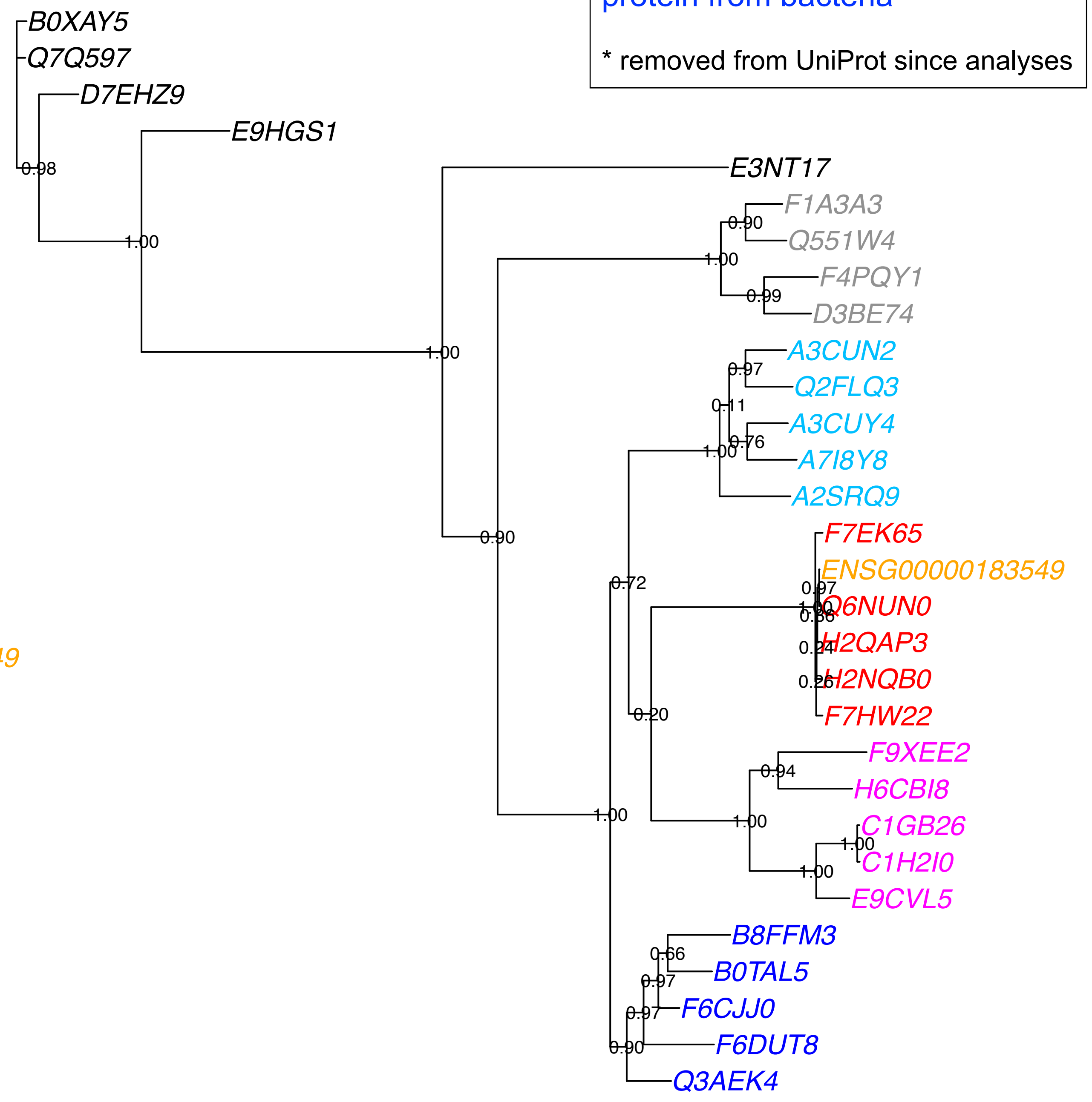

Phylogenetic tree excluding the contaminated genomes
